# Sexual Dimorphism of the Mouse Plasma Metabolome Is Associated with Phenotypes of 30 Gene Knockout Lines

**DOI:** 10.3390/metabo13080947

**Published:** 2023-08-15

**Authors:** Ying Zhang, Dinesh K. Barupal, Sili Fan, Bei Gao, Chao Zhu, Ann M. Flenniken, Colin McKerlie, Lauryl M. J. Nutter, Kevin C. Kent Lloyd, Oliver Fiehn

**Affiliations:** 1 West Coast Metabolomics Center, University of California Davis, Davis, CA 95616, USA; 2 Department of Chemistry, University of California Davis, Davis, CA 95616, USA; 3 Department of Environmental Medicine and Public Health, Icahn School of Medicine at Mount Sinai, New York, NY 10029, USA; dinesh.barupal@mssm.edu; 4 School of Marine Sciences, Nanjing University of Information Science and Technology, Nanjing 210044, China; 5 College of Medicine & Nursing, Dezhou University, Dezhou 253023, China; 6 The Centre for Phenogenomics, Toronto, ON M5T 3H7, Canada; flenniken@lunenfeld.ca (A.M.F.); colin.mckerlie@sickkids.ca (C.M.); lauryl.nutter@sickkids.ca (L.M.J.N.); 7 Lunenfeld-Tanenbaum Research Institute, Mount Sinai Hospital, Toronto, ON M5G 1X5, Canada; 8 The Hospital for Sick Children, Toronto, ON M5G 1X8, Canada; 9 Department of Surgery, School of Medicine, and Mouse Biology Program, University of California Davis, Davis, CA 95616, USA; kclloyd@ucdavis.edu

**Keywords:** animal models, lipidomics, mass spectrometry, physiology, complex diseases

## Abstract

Although metabolic alterations are observed in many monogenic and complex genetic disorders, the impact of most mammalian genes on cellular metabolism remains unknown. Understanding the effect of mouse gene dysfunction on metabolism can inform the functions of their human orthologues. We investigated the effect of loss-of-function mutations in 30 unique gene knockout (KO) lines on plasma metabolites, including genes coding for structural proteins (11 of 30), metabolic pathway enzymes (12 of 30) and protein kinases (7 of 30). Steroids, bile acids, oxylipins, primary metabolites, biogenic amines and complex lipids were analyzed with dedicated mass spectrometry platforms, yielding 827 identified metabolites in male and female KO mice and wildtype (WT) controls. Twenty-two percent of 23,698 KO versus WT comparison tests showed significant genotype effects on plasma metabolites. Fifty-six percent of identified metabolites were significantly different between the sexes in WT mice. Many of these metabolites were also found to have sexually dimorphic changes in KO lines. We used plasma metabolites to complement phenotype information exemplified for Dhfr, Idh1, Mfap4, Nek2, Npc2, Phyh and Sra1. The association of plasma metabolites with IMPC phenotypes showed dramatic sexual dimorphism in wildtype mice. We demonstrate how to link metabolomics to genotypes and (disease) phenotypes. Sex must be considered as critical factor in the biological interpretation of gene functions.

## 1. Introduction

Today, sexually dimorphic abnormal phenotypes are recognized in studies that focus on the pathophysiological consequences of genetic shift or reproduction [1,2,3,4,5,6,7,8,9,10,11]. Historically, extensive bias in using both sexes in animal studies resulted in misleading or incomplete conclusions [12,13,14,15,16]. In neuroscience, for example, studies in male rats, mice, monkeys, and other mammals outnumbered those in females by 5.5 to 1 [16]. In 2014, the U.S. National Institutes of Health issued a policy mandating the inclusion of female and male subjects in animal and cell research [12] and that sex be factored as a biological variable into research design, analysis and reporting [17]. Sexual dimorphism may be established by the action of hormones that are expressed in different amounts between the sexes [18]. These hormones may act differently on peripheral organs and thus impact a multitude of systems, including cardiovascular physiology, the immune system and nutrient absorption, and can be involved in complex diseases, such as type 2 diabetes, obesity and Alzheimer’s disease [19,20,21,22,23].

In general, sexual dimorphism has been observed in various classes of metabolites, such as branch-chain amino acids, phosphocholines, sphingomyelin and urea cycle metabolites [24,25,26,27]. Yet, the impact of gene dysfunction on metabolism has not been broadly studied. Specific genes may have pleiotropic effects on the metabolic systems of female and male cells, tissues, organs and organ systems [28,29,30,31]. To study the in vivo effects using a mammalian model, we obtained samples from KO mice generated by the International Mouse Phenotyping Consortium (IMPC), a global program linking 16 different research centres in 12 countries on five continents engaged in identifying the in vivo function of all protein-coding human gene homologs in the mouse genome. The IMPC uses standardized phenotyping protocols that generate ~1200 parameters. All IMPC material and data resources are publicly available for each of ~7000 KO lines phenotyped to date (https://www.mousephenotype.org/, accessed on 11 August 2023) [32], including 208 classified as continuous variables (Supplementary Data S1), some of which are metabolic traits, such as glucose tolerance and total plasma triacylglyceride content. For continuous traits in 2186 KO lines, 9% of phenotype parameters showed significant genotypic differences, of which 14% were sexually dimorphic [33].

Metabolomics is widely used in epidemiology and clinical research [34,35] to understand the mechanism of disease development [36,37]. Mass spectrometry is the dominant technology in targeted and untargeted metabolomics [38,39]. Version 4.0 of the Human Metabolome Database has records of more than 25,300 blood metabolites [40], but due to the chemical diversity of metabolites, individual analytic approaches can identify far fewer metabolites. Data sets from most individual metabolomic studies comprise 100–1200 metabolites [41,42,43]. We used multiple analytical methods to interrogate several metabolic pathways, including hydrophilic interaction liquid chromatography–mass spectrometry (HILIC-MS), gas chromatography–time-of-flight mass spectrometry (GC-TOF MS) and reverse-phase liquid chromatography–mass spectrometry (RPLC-MS) [44]. Mice are the most often used in vivo model to study the functions of genes compared to human orthologues. We here showcase how metabolomic data can be used to complement the existing IMPC data repositories to link sex, genotypes and phenotypes.

## 2. Materials and Methods

### 2.1. Mouse KO Production and Selection

All KO mouse lines were produced from C57BL/6N-derived targeted embryonic stem (ES) cells from the International Knockout Mouse Consortium (IKMC) using standard protocols at The Centre for Phenogenomics (TCP) in Toronto, Canada. All procedures involving mice were performed in compliance with the Animals for Research Act of Ontario and the Guidelines of the Canadian Council on Animal Care. TCP’s Institutional Animal Care Committee also reviewed and approved all procedures conducted on mice at TCP. Mice were subjected to phenotyping at TCP as part of the IMPC project using standardized protocols (IMPReSS, https://www.mousephenotype.org/impress) (accessed on 11 August 2023). Lithium heparin plasma of 30 KO lines (three mice per sex and gene) were selected from TCP’s bioarchive along with 40 corresponding C57BL/6NCrl WT mice (20 female, 20 male) [44]. All phenotype data are available from the IMPC website (https://www.mousephenotype.org/) [45]. All metabolomics data were obtained at the West Coast Metabolomics Center (WCMC) at UC Davis.

### 2.2. Metabolome Data Acquisition and Data Processing

Plasma samples were prepared by liquid-liquid extractions as published previously [44]. Pooled human plasma (BioIVT) samples were used as quality control (QC) samples in this study for each of the five platforms. Blanks and QC samples were prepared at the same time of mouse plasma sample extraction. All samples were randomized via miniX study design software [39], with one blank and one quality control (QC) sample between every 10 mouse plasma samples. In total, 22 QC samples were used for calculating the RSDs for each platform. Mass spectrometry data were acquired by using three untargeted assays: primary metabolites covering carbohydrates, amino acids, hydroxyl acids, and related compounds were analyzed using gas chromatography-time-of-flight mass spectrometry (GC-TOF MS, Leco Corporation, St. Joseph, MI, USA) [44]; complex lipids, such as phosphatidylcholines, ceramides, sphingomyelins, and triacylglycerides, were acquired using Vanquish UHPLC system with Q-Exactive HF mass spectrometry (Thermo Scientific, Waltham, MA, USA) [44]; and biogenic amines, such as carnitines, dipeptides, nucleosides, and modified amino acids, were analyzed using hydrophilic interaction chromatography–orbital ion trap mass spectrometry (HILIC-Q-Exactive MS/MS, Thermo Scientific, Waltham, MA, USA) [44]. Data on bile acids, steroids, and oxylipins were acquired by reversed phase LC separation followed by targeted analysis on a Sciex quadrupole-linear ion trap mass spectrometer (6500+ QTRAP MS/MS) [44]. The median RSD for compounds in QC samples was less than 20%. 827 metabolites remained in the dataset after removing deuterated internal standards, metabolites with at least 70% missing values or with an RSD > 50%, as well as removing doublet metabolites measured in more than one assay (Supplementary Data S2). HILIC-(ESI) MS data were normalized by the median value for each batch to remove batch effects. GC-TOF MS data were normalized using the SERRF method [44]. Data from other assays were not normalized because no batch effects were observed. Identified compounds were used for statistics if they were positively detected in more than 30% of all samples. Missing value treatment: For GC-TOF MS, the peak intensity values were automatically extracted from raw data (of the target *m*/*z* and target retention time) during the data processing procedure performed using GC BinBase [39], and missing values were automatically replaced by local noise. For other metabolomic assays, missing values were replaced in R by half of the minimum of the non-missing values in each mouse genotype. This procedure assumes that missing values are true negative signals, i.e., that such metabolite levels were too low to be detected by the mass spectrometer. Metabolites or phenotype data were discarded from analyses when the percentage of missing values was >70% of total number of mice in each sex group. Otherwise, missing phenotype data were replaced by the minimum of the non-missing values in each genotype mice. This procedure assumes that phenotype outputs have less variance than metabolite levels, but missing phenotype levels are likely to be minimal values.

### 2.3. Statistical Analyses

Because all mice were housed at TCP under identical standard operating procedures and were based on the same WT strain C57BL/6NCrl, and because plasma was collected from mice at similar ages (Supplementary Table S1), factors such as age, environment, and husbandry were constant and did not require statistical adjustments. Statistical analysis was performed using R 3.6.0. An overview on statistical workflows is given in Supplementary Figure S1.

Wildtype (WT) mice. Briefly, generalized linear regressions, specifically generalized-least-squares-fitted linear models (GLS) were used to test the role of sex as a variable (Y ~ sex) or as a covariate with adjusting body weight (Y ~ Sex + Weight). Errors in the GLS model can be correlated and/or have unequal variances. Compounds are deemed to be statistically significantly affected by sex at *p* < 0.05 or FDR < 0.05 after Benjamini-Hochberg false discovery rate adjustments. The same methods were used to analyze 208 IMPC phenotypes classified as continuous data parameters as extracted from the IMPC database. For graphic representation of results in boxplots or fold-change calculations, arithmetic means ± standard deviations were used.

KO mice: Briefly, we first studied the role of genotype alone, and subsequently we assessed the impact of genotype–sex interaction on plasma metabolite levels. Batch effect was not considered in analyses because all mice were raised and analyzed under identical conditions, and metabolomics data were acquired within one batch. For both investigations, two-way ANOVA was used for testing the statistical significance of genotype-sex interaction effect on each metabolite (*p* < 0.05). Two-way ANOVA is suitable to test how quantitative variables change depending on two categorical independent variables, like the combined impact of genotype and sex in this paper.

Body weight was not accounted for due to the very small difference between sex effect adjusted to body weight and sex effect without adjusting to body weight in WT mice, as well as the low number of mice per KO genotype and sex. A false discovery rate adjustment was not carried out on individual metabolite levels because the purpose of this study was to generate novel biochemical hypotheses rather than the discovery of biomarkers for diagnostic purposes. Also, evidence showed that there was only a small difference between sex effect with FDR correction and sex effect without FDR correction in wildtype. Instead, we combined all metabolites into classes to conduct set enrichment analysis with FDR correction using ChemRICH software [46] (see below). To test the genotype effect, a full model (Y ~ Genotype + Sex + Genotype: Sex) was compared with a null model (Y ~ Sex). For the genotype–sex interaction effect, the regression analysis compared the full model (Y ~ Genotype + Sex + Genotype: Sex) against the null model (Y ~ Genotype + Sex). Individual comparisons of genotype/WT for each sex were used to classify the genotype effect (Supplementary Figure S1c). If the plasma level of a specific metabolite was significantly different by the genotype effect and by genotype–sex interaction, then we specified whether effects were found only in females, only in males, in both sexes but in different directions of change, or in both sexes but with different effect sizes. Effect sizes were calculated by standardizing the associated arithmetic average estimate of each KO line to the mean of the corresponding WT mice. For other graphic representations in boxplots or fold-change calculations, arithmetic means ± standard deviations were used. For continuous phenotypic traits extracted from the IMPC database, the same methods were used by comparing a full model (Y ~ Genotype + Sex + Genotype: Sex) to a null model (Y ~ Sex) for assessing the genotype effect and by comparing the full model (Y ~ Genotype + Sex + Genotype: Sex) to a null model (Y ~ Genotype + Sex) for assessing the genotype–sex interaction effect, where the dependent variable is an adult or embryo phenotype parameter from IMPC.

Metabolite set enrichment statistics: Chemical Similarity Enrichment Analysis (ChemRICH) was used for finding differentially regulated clusters of metabolites [46]. ChemRICH defines different sets of molecules in metabolomics assays based on the Medical Subject Headings (MeSH) ontology term annotations and Tanimoto chemical similarity calculations. The PubChem identifier of each identified metabolite was mapped to MeSH terms in the ChemRICH database. For compounds that were not contained in the ChemRICH database, their MeSH terms were estimated using Tanimoto chemical similarity coefficients. According to PubChem CIDs, names, SMILES codes, and MeSH terms, metabolites were separated into non-overlapping chemical clusters. Then, Kolmogorov–Smirnov test was conducted on each cluster to assess the significance alteration compared to random distribution. A *p*-value less than 0.05 or FDR *p*-value less than 0.05 were used as criterion to determine the cluster significance.

Metabolite-phenotype correlation statistics: For WT mice, Spearman-rank correlations were used to calculate associations between 803 metabolites and 208 IMPC phenotypes. Spearman’s rank is a nonparametric correlation that measures the strength and direction of association between two ranked variables rather than their linear relationships. It is also more robust against outliers than Pearson’s correlation. ChemRICH was used for assessing metabolite clusters that were correlated with phenotypes [46]. Only metabolite-phenotype correlations with >14 data points per variable and per sex were subjected to ChemRICH analysis. *p*-values < 0.05 were used to determine cluster significance levels. 

## 3. Results

### 3.1. 827 Unique Metabolites Were Detected in Mouse Plasma

**Figure 1:**
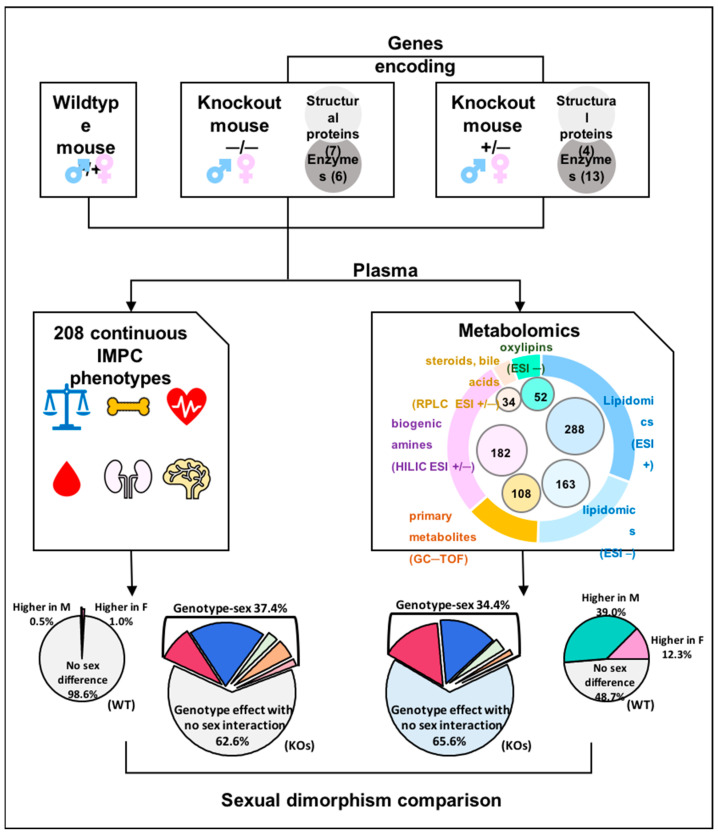
Study design and data analysis. Thirty gene knockout lines and corresponding wildtype controls were selected with phenotypic data available from the IMPC. Plasma samples of 220 mice were analyzed using five assays, including three untargeted metabolomic profiling and two targeted data acquisition methods.

Two-hundred and twenty mice (110 female and 110 male) on the C57BL/6NCrl background produced and phenotyped by The Center for Phenogenomics (TCP, Toronto, ON, Canada) as part of the IMPC project were used for this study. Approximately 30% of KO lines analyzed by the IMPC are embryonic lethal [47], for which test cohorts of heterozygous KO mice were used for adult phenotyping. Blood plasma was obtained at the end of the in vivo phenotyping pipeline. The metabolic impact of null mutations was tested on 17 adult heterozygous lines and 13 adult homozygous lines (Figure 1) using six mice (three female and three male) of each KO line. Control samples were collected from 40 (20 female and 20 male) co-housed sex- and age-matched C57BL/6NCrl WT mice over the same time period as the KO mice (Supplementary Table S1). KO lines of genes coding for structural proteins (11 of 30), metabolic pathway enzymes (12 of 30) and protein kinases (7 of 30) were selected to assess their impact on blood metabolic phenotypes. Among those genes, 16 gene KOs were used as models for human diseases (by similarities of annotation and orthology) (Supplementary Table S1). Using previously established IMPC criteria, phenotypes of these disease mouse models overlapped with human diseases that harbor mutations in the mouse orthologous genes [47]. We acquired comprehensive metabolomic data using a total of 20 μL lithium heparin plasma per mouse for three untargeted metabolomics assays and an additional 50 μL lithium heparin plasma to target low-abundance bile acids, steroids and oxylipins [44]. In total, 827 unique metabolites identified using these five mass spectrometry assays were utilized for sexual dimorphism assessment (Supplementary Data S2), detecting 21 bile acids, 13 steroids, 52 oxylipins, 108 primary metabolites, 451 complex lipids and 182 biogenic amines. The assays showed little overlap of compounds (Figure 1, Supplementary Data S2), proving their complementary nature and utility.

### 3.2. Sexual Dimorphism in Wildtype (WT) Mice

**Figure 2:**
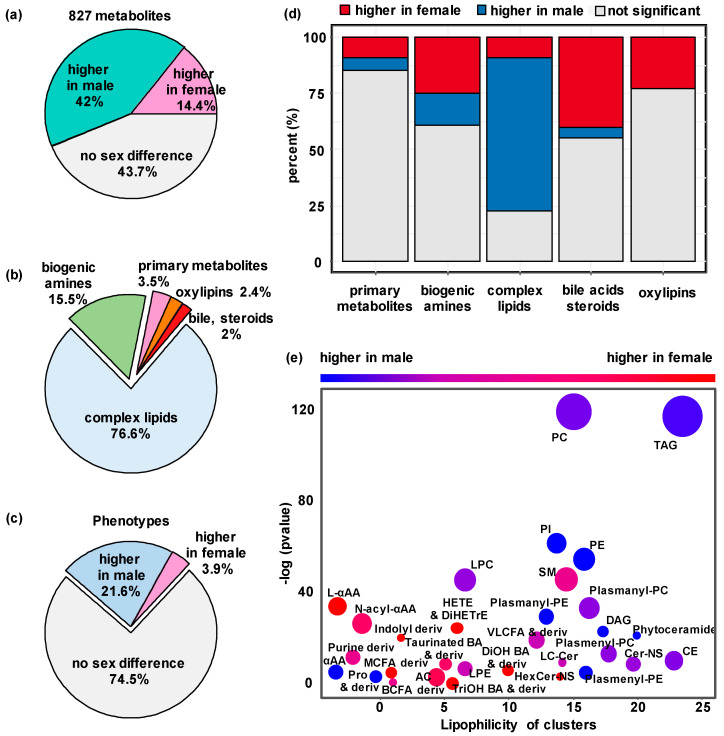
Sex differences in wildtype mice. (**a**) Proportion of metabolites that were significantly affected by sex in 40 wildtype mice *(n* = 20 males and 20 females; generalized linear model at *p* < 0.05). (**b**) Distribution of sex-affected metabolites across five metabolic assays (*p* < 0.05). (**c**) Proportion of continuous IMPC phenotypes that were significantly affected by sex in 40 wildtype mice (*p* < 0.05). (**d**) Metabolites affected by sex per metabolomics assay (*p* < 0.05). (**e**) Chemical Similarity Enrichment Analysis between male and female wildtype mice (dot size proportional to number of metabolites.).

The differences in metabolomic data were first assessed between 20 female and 20 male WT mice. Fifty-six percent of all plasma metabolites displayed sexual dimorphism using a generalized linear model at *p* < 0.05 (Supplementary Data S3). The levels of 337 of the 805 metabolites (41.9%) were increased in male mice compared to females, and 116 of the 805 metabolites (14.4%) were more abundant in female than in male mice (Figure 2a). With a more stringent criterion at FDR < 0.05, this analysis only slightly changed the proportion of significant metabolites from 56.3% to 51.8%, with 39.3% plasma metabolites more abundant in male mice and 12.6% in female mice (Supplementary Figure S2a). Using chemical similarity enrichment statistics by ChemRICH impact plot 46, we found 31 classes of compounds that were enriched via a direct comparison of the two sexes (Figure 2e). Most complex lipids showed higher plasma levels in male mice than in females (Figure 2b,d,e), for example, with higher abundances of triacylglycerides (TAG), phosphatidylcholines (PC) and phosphoinositides (PI) in WT males. Many other metabolites were found at higher levels in female mice than in males (Figure 2d). Within the biogenic amines, some metabolite classes, such as acylcarnitines, indole derivatives and L-alpha-amino acids, showed sexual dimorphism with higher levels detected in WT female mouse plasma (Figure 2e). While the sexual dimorphism is dominated by complex lipids, the contribution of bile acids and oxylipins seem to exceed that of primary metabolites in terms of their proportions in each platform (Figure 2d). When analyzing previously reported IMPC phenotype data 47, we also found a strong sexual dimorphism in in vivo phenotypes of these WT mice (Figure 2a,c), with similarly more variables significantly greater in male mice compared to females. Overall, plasma metabolites levels were so different that perfect discrimination between the sexes was achieved using PLS-DA multivariate statistics (Supplementary Figure S3).

Metabolite levels may be impacted by total body weight. Hence, the sexual dimorphism of metabolites could be attributed to these known differences between the sexes. We therefore conducted a secondary analysis by adjusting metabolite levels to body weight at the time of blood collection. After adjusting to body weight at an FDR < 0.05, 51.3% of all metabolites showed sexual dimorphism (39.0% of metabolites higher in male mice and 12.3% higher in females, Supplementary Figure S2d). For comparison, 0.5% of the 208 IMPC-measured continuous phenotypic variables (Supplementary Data S4) were higher in wildtype male mice after body weight adjustment and FDR correction, and 1.0% of the phenotypes were increased in female mice (Supplementary Figure S2f). These results showed that differences in body weight impacted the sex differences in phenotype data but not in plasma metabolite levels.

Table 1 shows 20 individual examples of metabolites that were significantly different between WT female and male mice. Metabolites were chosen based on metabolic classes, significance levels and fold-change. Most of these metabolites were also found to be affected by KO mutations, except for a single phosphatidylcholine membrane lipid (PC 40:4). Many of these compounds also showed sexual dimorphism in KO lines.

Based on studies of human cohorts, including men and women, blood metabolite levels usually do not show large differences [48], often displaying changes of less than 30% in human-genome-wide association studies when analyzing the differences between sexes [49,50]. In this study we found more than 18.5% of the 805 metabolites showed a greater than two-fold sex difference in WT mice (Supplementary Data S3). The plasma levels of sex hormones were expected to be significantly different between the sexes, with testosterone only detected in the plasma of WT male mice. Progesterone was found at 2.5-fold higher levels in WT female mice due to their role in the estrous cycle, but it was also present in male mice as a crucial intermediate in the production of other endogenous steroids.

A similar concentration ratio between sexes has also been reported in humans [51]. Yet, many other compounds were not well known to be differentially present in the sexes. For example, the gut microbial metabolite trimethylamine-N-oxide (TMAO) was found at 4-fold higher levels in female than in male mice (Table 1, Supplementary Data S3). Different levels of circulating TMAO have also been observed in humans [52]. Similarly, specific microbially transformed secondary bile acids, like glycocholic acid and tauroursodeoxycholic acid, were also found at higher levels in plasma from female mice, indicating a potentially differential impact of the gut microbiome on the sexes. A range of arachidonyl lipid mediators, such as the oxylipin 11,12-epoxyeicosa-5,8,14-trienoic acid (11,12-EpETrE), were detected in increased concentrations in female compared to male mice (Table 1), as well as 8,9-epoxyeicosatrienoic acid (8,9-EpETrE) and 8,9-dihydroxy-5Z,11Z,14Z-eicosatrienoic acid (8,9-DiHETrE) (Supplementary Data S3). Conversely, the most significantly elevated plasma metabolites in male mice mainly belonged to polar and neutral lipid species (Table 1, Figure 2e), including diacyl- and monoacylphosphatidylcholines (PC and LPC), phosphatidylethanolamines (PE) and neutral fats (triacylglycerides (TAG)). An adjustment to whole body weight differences between the sexes did not change this finding. Higher plasma levels of both membrane phospholipids and fats are partly explained by higher levels of circulating lipoproteins in male mice. Indeed, high-density lipoprotein (HDL) cholesterol and non-HDL cholesterol were determined to be 37% and 7% higher in male mice, respectively (Supplementary Data S4), along with total plasma TAGs measured by the IMPC clinical chemistry protocols in concordance with our LC-MS/MS determination of significantly higher individual plasma TAG levels in male mice. 

### 3.3. Sexual Dimorphism in Mouse Gene Knockout (KO) Lines

Two-way ANOVA revealed sexual dimorphism in 30 KO mouse lines. We found that more than 260 metabolites had significant genotype effects with more than two-fold differences in plasma levels (Supplementary Table S2), such as a three-fold change in plasma adenosine level in Sra1−/− (steroid receptor agonist 1) mice or 12(13)-Ep-9-KODE with a greater than three-fold change in Npc2+/− (NPC intracellular cholesterol transporter 2) mice compared to WT controls (Supplementary Data S5). When performing multivariate analyses (PLS-DA) on all genotypes by sex, the strongest metabolic discriminant vector 1 was always driven by sexual dimorphism, with genetic effects explained by discriminant vector 2 (Supplementary Figure S4).

**Figure 3:**
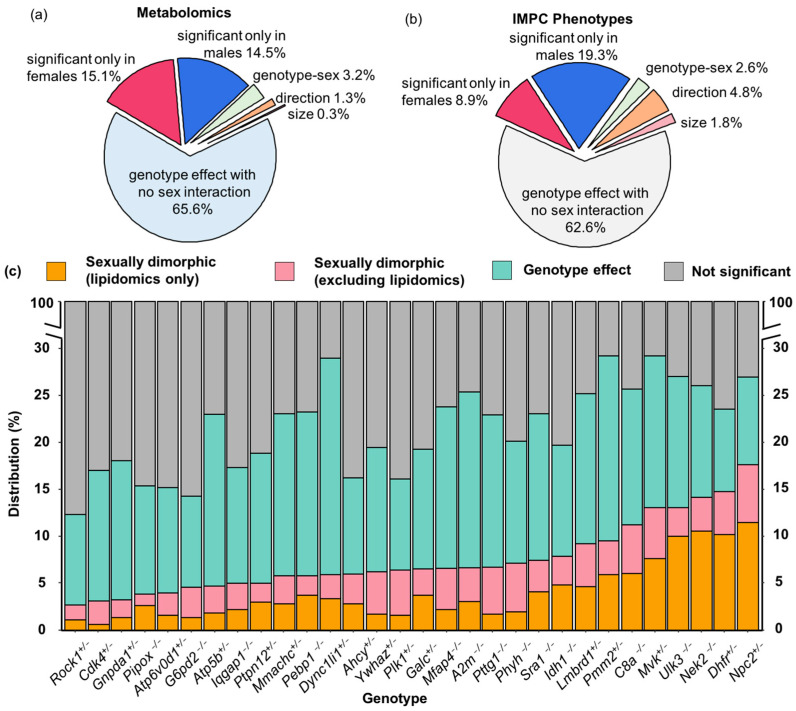
Overall genotype effect with sex interaction on metabolomics/phenotype data of 30 mouse knockout lines, comparing 40 C57BL/6NCrl controls to six mice per KO line. Two-way ANOVA at *p* < 0.05. (**a**) Proportion of significant genotype and sex interaction effects on metabolites. (**b**) Proportion of significant genotype and sex interaction effect on the IMPC phenotypes. (**c**) Distribution of metabolites that were altered by genotype effect and genotype–sex interaction effect for each KO line.

Next, we explored if the plasma metabolite levels of the KO lines were differentially influenced in male and female mice (Figure 3). All mice were housed at The Center for Phenogenomics under identical conditions using standard operating procedures and on the same inbred strain background, C57BL/6NCrl [45]. Hence, we excluded environmental and husbandry effects that were unrelated to genotype–sex interactions. We conducted two-way ANOVA tests to assess the interaction effect of genotype and sex on all 208 continuous phenotypes reported by the IMPC (Supplementary Table S3), and on our metabolome data for the 30 KO lines in this study. IMPC phenotype data showed that 26.3% of the 4756 comparisons across the 30 KO lines had significant phenotype differences (5.0% from body weight measured at different time points; Supplementary Data S6). Among those differences, 37.4% of the phenotypes were found to be sexually dimorphic (6.7% from body weight measured at different time points; Figure 3b, Supplementary Data S6). For metabolite levels that were significant at the genotype level alone but not for genotype–sex interaction effect, effects were classified as ‘genotype effect with no sex difference’ in Figure 3.

Our metabolomic data identified 21.5% among the 23,698 KO lines/WT comparison tests were significant at *p* < 0.05. Similarly, about one third of metabolomic changes showed significant genotype–sex interactions (Figure 3a). Most changes were based on alterations in one sex but not the other. While both phenotype (Figure 3b) and metabolome analyses (Figure 3a) showed that most significant differences were true for both sexes and at similar magnitude and direction, phenotype analyses had a higher percent of traits that were significantly different from WT mice in both sexes but either with changes in different directions or with different effect sizes between the two sexes (Figure 3b). In comparison, metabolome analyses showed that the proportion of metabolites different from WT in only one sex was slightly higher with a lower proportion of metabolites showing opposite directions and different effect sizes in the two sexes (Figure 3a,b). For metabolome data, the degree of sexual dimorphism was more pronounced in some KO lines than in others (Figure 3c). For example, 26.9% of all plasma metabolites were altered in Npc2+/− mice, but plasma levels of almost 64.8% of those compounds were sexually dimorphic (Figure 3c). Similarly, 44.8% of all significantly genotype-affected metabolites in the Mvk+/− (metabolic enzyme mevalonate kinase) mice were found to be sexually dimorphic (Figure 3c).

Initially we hypothesized that many genes might be so distant from enzymatic functions that no overall changes of metabolic phenotypes would be detected in mouse KO plasma. However, we identified at least a few metabolites that were differently affected between the sexes compared to WT mice in each of the 30 KO lines (Figure 3c). Each gene KO showed specific metabolites that were differentially regulated between male and female mice, ranging from 3 to 17% of the annotated metabolome (Figure 3c). For example, the least affected KO mouse line was Rock1+/−, coding for a gene involved in the regulation of cell motility, cell cycle and cell adhesion, with about 12.3% of the metabolome found to be changed in Rock1+/− mice compared to WT controls, of which 21.7% showed sexual dimorphism. A similarly small number of metabolic changes were found in Ckd4+/− mice, coding for another kinase involved in regulating cell cycle and tumorigenesis (Figure 3c). As expected, genes that directly targeted metabolic enzymes or overall organ development had a stronger impact on the plasma metabolome with changes of up to 29% in all identified compounds. Examples of KO lines with genes targeting metabolism are Pmm2+/−, coding for a phosphomannomutase involved in protein glycosylations, the cholesterol transporter Npc2+/−, and Idh1−/− that codes for isocitrate dehydrogenase 1. Similarly, a higher number of metabolic differences were found in gene KO lines involved in organ development, such as the sonic hedgehog unc-51-like kinase 3 (Ulk3−/−) that is involved in embryonic development (Figure 3c).

### 3.4. Genotype-Sex Interactions on Lipids in Dhfr+/−, Npc2+/−, Nek2−/− and Sra1−/− Lines

Three genes, Dhfr, Npc2, and Nek2, had the highest proportion of lipids with genotype–sex interaction effects (Figure 3c and Supplementary Data S5). Two of these genes code for enzymes that are known for their impact on cell division and therefore are actively studied in cancer research. DHFR (dihydrofolate reductase) is important in nucleoside biosynthesis [53], and NEK2 (NIMA-related kinase 2) is a centrosome kinase involved in Wnt-signaling pathways [54,55]. By altering the rate of cell division, both enzymes can act on lipid metabolism. More lipids were impacted in Nek2−/− females than in males, while divergent changes in plasma lipids levels were observed in Dhfr+/− male and female mice (Supplementary Data S5).

**Figure 4:**
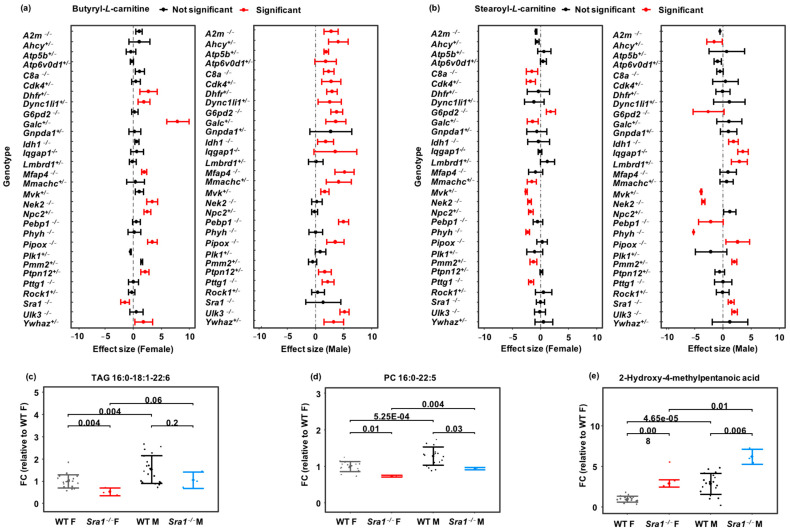
Examples of metabolites affected by genotype–sex interaction of 30 mouse knockout lines. Comparison of 40 C57BL/6NCrl control mice versus *n* = 6 mice per knockout line. Two-way ANOVA followed by individual comparisons using a generalized linear model. (**a**,**b**) Plasma acylcarnitines in all genotypes. Error bars represent ranges of standardized effect sizes. (**c**–**e**) Plasma metabolites in Sra1−/− mice versus controls. Standardized fold-changes normalized to wildtype mice with error bars ±1 s.d.

The gene that had the highest fraction of sexually dimorphic plasma lipid levels was Npc2. Overall, 26.9% of all plasma metabolites were affected in Npc2+/− mice (Figure 3c). Of these metabolites, 51.2% were detected by the lipidomics assay but were confined to Npc2+/− female mice (Supplementary Data S5). Npc2 encodes the intracellular cholesterol transporter 2, regulating the transport of cholesterol to the perimeter membrane of late endosomes, to become available for transporting payloads to mitochondria, leading to cholesterol accumulation in lysosomes. In humans, a defect in NPC2-related cholesterol trafficking leads to the ultimately fatal Niemann–Pick Type C2 (NPC2) disease, an autosomal recessive complex lipid storage disorder [56]. Therefore, we expected to detect primary changes in lipid signatures. However, differences in the mitochondrial fatty acid transport molecules butyryl-carnitine and stearoyl-carnitine were only found in female Npc2+/− mice and not in male Npc2+/− mice (Figure 4a,b).

Interestingly, both acylcarnitines were found to be dysregulated in many KO lines, often in a sexually dimorphic manner (Figure 4a,b). Since mitochondrial fatty acid oxidation is a major contributor to overall energy usage, such data may enable a better understanding of the impact of gene dysfunction in human disease phenotypes. Lipids were also much more affected in Sra1−/− female than Sra1−/− male mice. For example, many triacylglycerides and phosphocholines were significantly down-regulated in Sra1−/− female mice, but not always in males (Figure 4c,d). The bacterial leucine-derived metabolite 2-hydroxy-4-methylpentanoic acid [57,58] was higher in the plasma of both Sra1−/− male and female mice (Figure 4e). This observation shows again that gene KO mutations can have metabolic effects that involve the microbiome. The Sra1 gene encodes the steroid receptor RNA activator protein and is involved in breast tumorigenesis and tumor progression. Our metabolomic results showed that not only triacylglycerides but also mitochondrial acylcarnitines, oxylipins and sphingomyelins were differentially regulated in Sra1−/− female and male mice. Several oxylipins that can act as potent physiological mediators [59,60] were significantly upregulated, including 8,9-DiHETrE and 11,12-EpETrE (in female Sra1−/− mice at *p* < 0.05), 11-HETE (in Sra1−/− male mice at *p* < 0.01) and 15-HETE (fold-change = 1.4 and 1.9 in Sra1−/− female and male mice, respectively), while three other oxylipins were downregulated, including 15-HEPE and 15-KETE (in Sra1−/− female mice at *p* < 0.05) as well as 9,10-e-DiHO (in Sra1−/− male mice at *p* < 0.01; Supplementary Data S5).

### 3.5. Phyh−/−, Npc2+/− and Mfap4−/− Impact Plasma Lipid and Peptide Metabolism

Some genes are known for their implicit contribution to human diseases, such as Phyh, Pmm2 and Npc2, while many others may not directly lead to a specific disease but may participate in the pathophysiology of some diseases. However, the influence of their dysfunction on metabolism is not fully understood. Phyh, a major player in Refsum disease, encodes phytanoyl-CoA hydroxylase, which is responsible for breaking down phytanic acid in the alpha-oxidation pathway. Indeed, our metabolomic results (Supplementary Data S5) showed the differential regulation of the plasma levels of 2-hydroxylated (branched) fatty acids, such as 2-hydroxy-3-methylbutyric acid (fold-change = 1.6 in Phyh−/− male at *p* < 0.001) and 2-hydroxy-4-methylpentanoic acid (fold-change = 2.4 in Phyh−/− female at *p* = 0.01); and the carnitine transport forms of 3-methyl-fatty acids, such as 3-hydroxyisovaleroylcarnitine (fold-change = 0.8 in Phyh−/− female at *p* = 0.02), isovaleryl-carnitine (fold-change = 0.8─0.9 in both sexes with *p* = 0.2) as well as derivatives of 3-methyl-fatty acid structures, including valine (fold-change = 1.4 in Phyh−/− female at *p* = 0.02), isovaleryl-glycine (fold-change = 1.8 in Phyh−/− male at *p* = 0.04) and valine-dipeptides. The consequential genotype effect also exists for other metabolites, such as decreased plasma levels of straight-chain fatty-acyl carnitines. Plasma prostanoids were upregulated in Phyh−/− mice, including progesterone (fold-change = 2.2 in Phyh−/− female at *p* = 0.006) and PGF3alpha (fold-change = 2.8 in Phyh−/− male at *p* < 0.001), and plasma bile acids were impacted by genotype–sex interaction, including tauroursodeoxycholic acid (fold-change = 0.3 in Phyh−/− female at *p* = 0.003) and glycocholic acid (fold-change = 5.7 in Phyh−/− male at *p* = 0.02).

**Figure 5:**
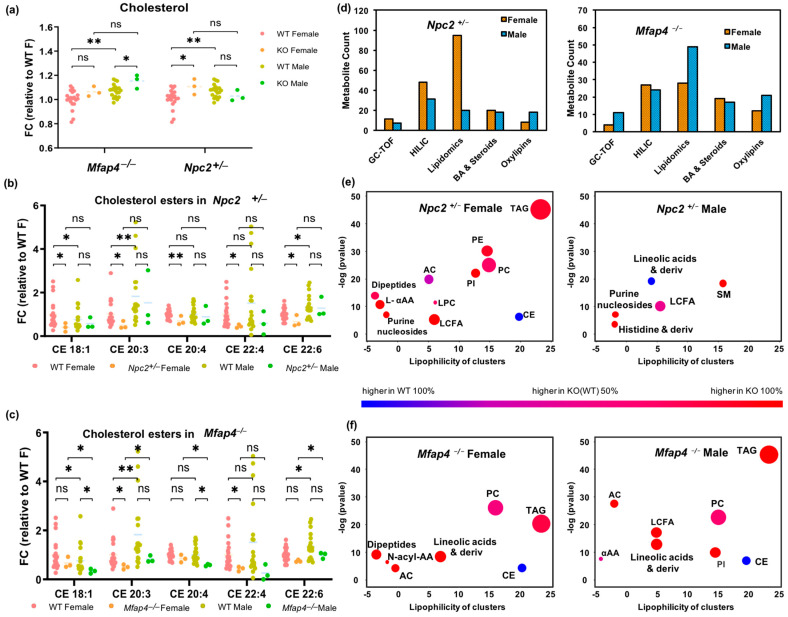
Comparison of sexually dimorphic plasma metabolites for Npc2+/− and Mfap4−/− mice versus C57BL/6NCrl control mice. Significance levels * *p* < 0.05; ** *p* < 0.01, ns not significant. (**a**) Plasma cholesterol levels in Npc2+/− and Mfap4−/− mice versus controls. (**b**) Plasma cholesterol ester levels in Npc2+/− mice versus controls. (**c**) Plasma cholesterol ester levels in Mfap4 −/− mice versus controls. (**d**) Number of significant metabolites for Npc2+/− and Mfap4−/− mice versus controls. (**e**) Chemical set enrichment plots for Npc2+/− mice versus controls. (**f**) Chemical set enrichment plots for Mfap4−/− mice versus controls. Dot sizes are proportional to number of metabolites per chemical set.

Because homozygous null mutations in Npc2 are lethal, the viable heterozygotes were used for this study (Supplementary Table S1). Human NPC2 is mainly expressed in the lungs, thyroid and gall bladder [61]. In addition to Niemann–Pick Type C2 (NPC2) disease, NPC2 is involved in chronic obstructive pulmonary disease (COPD) [62]. Microfibril-associated protein 4 coding gene Mfap4 also has high expression in the lungs and gall bladder [61] and is upregulated in COPD [63]. The MFAP4 protein has binding specificities for both collagen and carbohydrates. We report here the first metabolomic effects of these two genes (Figure 5). Because cholesterol trafficking is impaired in Npc2+/− mutants, we investigated if lower plasma levels of cholesterol esters (CE) were observed. Indeed, a range of both plasma cholesterol esters and free cholesterol were found significantly altered, specifically in female mice (Figure 5a,b). Similarly, significant differences in both CE lipids and free cholesterol were found in Mfap4−/− mice, but male mice were more affected (Figure 5a,c). For both genotypes, the largest number of metabolic changes were observed in lipid metabolism, extending to steroids, bile acids, oxylipins (Figure 5d), and a range of phospholipids and neutral lipids that showed great effects in metabolite set enrichment statistics (Figure 5e–f), with notable differences between Npc2+/− female and male mice. Effects on hydrophilic metabolites were found for amino acids in both sexes of Mfap4−/− and Npc2+/− genotypes, but not in other metabolic modules, such as dipeptides, carbohydrates, or nucleosides (Supplementary Data S5). In comparison, sexually dimorphic metabolic alterations were much more prevalent in Npc2+/− mice (Figure 5e) than that in Mfap4−/− mice (Figure 5f). In combination, these results suggest that genes participating in the same disease (e.g., Mfap4 and Npc2 in COPD) may execute major effects on similar metabolic modules but, in addition, may also exert specific sexually dimorphic influence over other metabolic phenotypes.

### 3.6. Metabolic Alterations in Idh1−/− Mice

**Figure 6:**
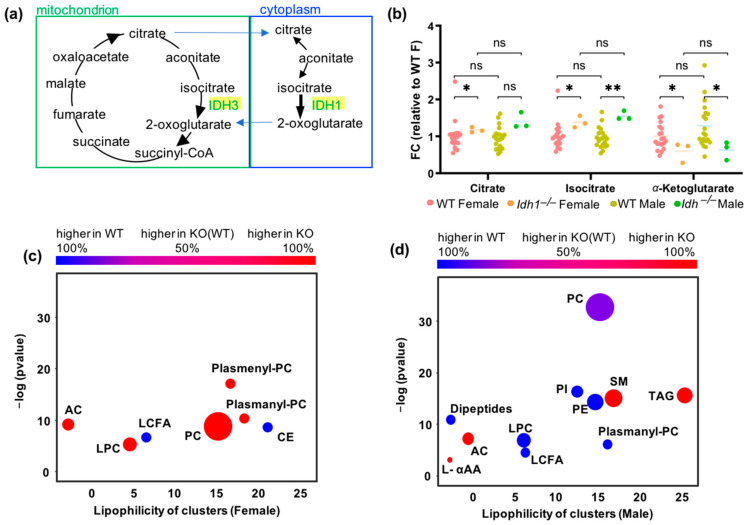
Sexual dimorphic alteration of plasma metabolites in six Idh1−/− mice versus 40 C57BL/6NCrl control mice. Significance levels given as * *p* < 0.05; ** *p* < 0.01, ns not significant. (**a**) Function of isocitrate dehydrogenase (IDH) in cytoplasm and mitochondria. (**b**) Changes of isocitrate, citrate and 2-oxoglutarate (α-ketoglutarate) plasma levels. (**c**) Chemical set enrichment of plasma levels in female Idh1 −/− mice versus controls. (**d**) Chemical set enrichment of plasma levels in male Idh1 −/− mice versus controls. Larger dots indicate a higher number of metabolites per chemical set.

The enzyme isocitrate dehydrogenase 1 (IDH1) catalyzes the cytoplasmic oxidative decarboxylation of isocitrate to α-ketoglutarate with an identical reaction performed by IDH3 in the tricarboxylic acid cycle (TCA cycle, Figure 6a). Therefore, the overall effect of the Idh1−/− allele might not be detectable on plasma concentrations of these metabolites due to a compensatory effect of the corresponding mitochondrial transporters and mitochondrial IDH3 enzyme (Figure 6a). The plasma levels of the substrates of IDH1-catalyzed reactions, citrate and isocitrate, showed significant increases in Idh1−/− mice (isocitrate with 1.4–1.6 fold-changes at *p* < 0.04 for both sexes, citrate with 1.3–1.4 fold-changes at *p* = 0.04 in female mice, and *p* = 0.07 in male mice; Figure 6b). Consequently, plasma levels of the reaction product α-ketoglutarate were downregulated in both sexes with 0.5–0.6 fold-changes at *p* < 0.04 (Figure 6b). This KO line is therefore a prime example of a direct match of the immediate biochemical reaction in the cell and plasma metabolite levels. Downstream metabolite levels, such as plasma glutamate, were also found to be significantly decreased in Idh1−/− male mice (fold-change = 0.76 at *p* = 0.03) (Supplementary Data S5) but not in female mice. Further pleiotropic effects of Idh1 KO mutation were found in both sexes alike as well as in a sexually dimorphic way. For example, in both sexes, Idh1−/− mutants showed set enrichment increases in acylcarnitines and decreases in free long-chain fatty acids. Yet, in Idh1−/− male mice, we found significantly decreased plasma levels of phosphatidylinositols, phosphatidylethanolamine and phosphatidylcholines as well as increased levels of triacylglycerides (Figure 6d), while in Idh1−/− female mice phosphatidylcholines levels were increased, and levels of cholesteryl esters were decreased (Figure 6c).

### 3.7. Sexual Dimorphism in Metabolite-Phenotype Associations

Sixteen out of thirty mouse KO lines are used as models for human diseases. Those genes showed overlap between phenotypes observed in human diseases and phenotypes observed in mouse KOs for orthologous genes (Supplementary Table S1). The relationships between plasma metabolite and phenotype parameters/parameter series may provide additional information to understand the gene functions and disease etiology. Due to the limited number of mice per sex per KO line, the associations were only performed in WT mice where strong sexual dimorphism was also observed.

To assess the magnitude of the associations between metabolites and IMPC phenotypes, Spearman correlation analysis was performed in wildtype mice (Supplementary Data S7). A total of 803 metabolites were correlated to 208 phenotypes, yielding 167,024 metabolite–phenotype spearman rank correlations in wildtype mice. A total of 20,435 correlations were significant at *p* < 0.05 (12.2%). Among those correlations, 9136 were only significant in females, while 10,392 were only significant in males. Only 742 correlations (3.6%) were significant in both sexes and in the same direction, indicating an overwhelming sexual dimorphism in metabolite–phenotype correlations. A small fraction of 165 metabolite–phenotype correlations were even statistically significant with opposite directions between the two sexes. For example, TAG 16:0–18:0–22:0, a triacylglyceride with three saturated free fatty acid chains, was negatively correlated with the phenotype ‘center average speed’ (one parameter in open field tests for anxiety) in wildtype females (r = −0.71 at *p* = 0.0007) but turned positive in wildtype male mice (r = +0.74 at *p* = 0.0002) (Supplementary Data S7). Pre-pulse inhibition (PPI) is the suppression of an acoustic startle reflex (ASR) to an intense stimulus when a weak pre-pulse stimulus precedes the startle stimulus. A reduction in PPI is thought to reflect the dysfunction of sensorimotor gating, which normally suppresses excessive behavioral responses to disruptive stimuli [64]. Several clinical studies linked human disorders with impaired PPI, including schizophrenia, Huntington’s disease, fragile X syndrome and autism. Phosphatidylcholine PC 40:4 was positively correlated with % PPI2 in female WT mice but was negatively correlated in male WT mice (Supplementary Data S7). Several other PCs/Plasmanyl-PCs showed sexual dimorphism correlations with other PPI phenotype parameters (Supplementary Data S7, Supplementary Figure S5a).

**Figure 7:**
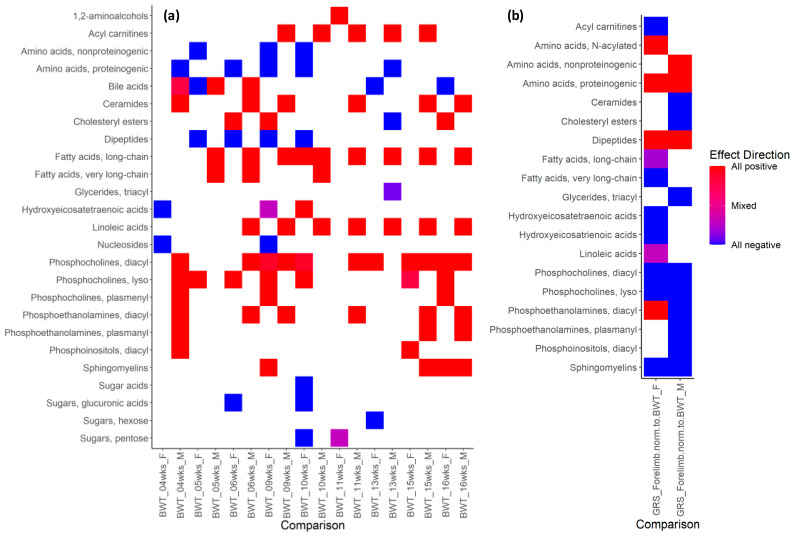
Heatmap of ChemRICH set enrichment clusters for female (F) and male (M) wildtype mice, calculated from Spearman rank correlations of metabolite versus IMPC phenotypes. (**a**) Clusters of significant **metabolite–body weight** phenotype associations (week 4–16). (**b**) Clusters of significant **metabolite–grip strength** phenotype associations, adjusted to body weight. Grip strength measures the neuromuscular function as maximal muscle strength of forelimbs and combined forelimbs and hind limbs. Average value from three trials were normalized to body weight. Spearman correlation with *p* < 0.05. N > 14 per sex for each metabolite and phenotype. Red: positive correlations; blue: negative correlations. (see Supplementary Data S7).

Because every single phenotype/metabolite association might be caused by random chance, we accumulated all data in more robust chemical enrichment statistics (ChemRICH) analysis. Based on 10 phenotype pipelines (Supplementary Data S7), metabolites were clustered by ClassyFire [65] and subjected to ChemRICH analysis to associate clusters of metabolites with specific phenotypes. As expected, metabolomic measurements were correlated with blood chemistry data acquired by the IMPC (Supplementary Figure S5b). For example, ‘metabolomic’ triacylglycerides and hexoses were positively correlated with IMPC triglycerides and glucose in both sexes (Supplementary Figure S5b). Dramatic sexual dimorphism in metabolite cluster–phenotype correlations were found in each of the 10 phenotype pipelines. For example, body weight (Figure 7a) was highly positively associated with acylcarnitines and linoleic-acid-derivatives as well as long-chain fatty-acid-derivatives in males but not in females, starting from week 5. Conversely, lysophosphatidylcholines were positively associated with body weight in females from five weeks onward, but not in males. Similarly, dipeptides and proteinogenic amino acids were negatively associated with body weight in females from 5 to 10 weeks but not in males (Figure 7a). Since body weight includes muscle mass, we also studied the association of plasma metabolites with grip strength, normalized to body weight (Figure 7b). While several compound classes (proteinogenic amino acids, dipeptides, sphingomyelins and phosphocholines) were found correlated in both sexes with forelimb grip strength, there was also remarkable sexual dimorphism. For example, plasma nonproteinogenic amino acids in males were positively associated with forelimb grip strength but not in females. Conversely, in females, N-acylated amino acids were found to be positively associated with forelimb grip strength but not in males. Similarly, sexual dimorphism was found for mitochondrial acylcarnitines and in linoleic-acid-derivatives and other compound classes.

All metabolite-phenotype heatmaps are given in Supplementary Figure S5 for acoustic startle/pre-pulse inhibition, clinical chemistry DEXA body composition, electrocardiograms, hematology, heart weight, intraperitoneal glucose tolerance and open field test (anxiogenic area). 

## 4. Discussion

We found that overt phenotypes measured by the IMPC showed ~25% of all phenotypic measures to be sexually dimorphic, while more than 56% of all plasma metabolites were significantly different between males and females. This finding indicates that sex has a major impact on many different metabolic pathways in mice [66,67]. Mice are widely used in pre-clinical research for curing human diseases. Often, metabolites can be found to be associated with other metabolites, genes, proteins and phenotypes. Excluding sex as a factor may hamper the disease biomarker discovery, disease mechanism interpretation and novel therapeutics development. Moreover, FDR correction and body weight adjustment did not influence the statistical results of sexual dimorphism in metabolomics data. We therefore conclude that sex differences in plasma metabolite levels were not simply explainable by differences in body weight. Oxylipins, indolyl derivatives, bile acids, amino acids and sphingomyelins were all increased more in female mice than in males, pointing to differences in the use of metabolites with regulatory roles that are well known for sphingomyelins [68], oxylipins [69], or indoles [70] and bile acids [71]. Even metabolites like TMAO that are clearly formed by gut intestinal microbes showed different levels in male and female mice, the level of which was also reported for rats and humans [25,72,73]. TMAO might act by stabilizing proteins as a “chemical chaperone” in the endoplasmic reticulum (ER) [74]. Levels of TMAO are associated with cardiovascular risk [74,75] and likely other diseases [76,77,78,79,80,81]. This finding shows that levels of metabolites produced by many routes (including by different microbiomes) are sex-dependent, suggesting that metabolomic data might best be interpreted differently between the sexes. Similar to TMAO, we found EpETrEs (also called EETs) to be sexually dimorphic. EETs are derived from arachidonate and have opposite effects to TMAO with respect to vasodilatory impact and other cardioprotective effects [82,83,84]. Lipid mediators, including EETs, are important actors in the regulation of a range of physiological parameters, such as blood pressure [69,85], but are not generally known to have sexually dimorphic levels in humans. Hence, such sexually dimorphic metabolites might contribute to the well-known differences in cardiovascular risk in men compared to women [86]. In addition, we found many lipid classes to be elevated in WT male mice such as membrane lipids, cholesteryl esters and triacylglycerides, whereas signaling lipids, like phospho-sphingolipids, were elevated in female mice. Similar to TMAO and EETs, such findings have implications for health effects as the excessive accumulation of TAG are known to be associated with a range of diseases, including hepatic steatosis and non-alcoholic fatty liver disease [87]. Indeed, in humans, men also have higher TAG blood levels [88] and higher VLDL lipoprotein particle levels than women [89], which is highly correlated with the higher risk of atherosclerotic cardiovascular disease in men [90].

On the other hand, though sexual dimorphism was also observed in many human healthy cohort studies, the sex effect on metabolism can differ greatly between humans and mice as well as between different human cohorts [49,50,91,92,93]. Sexual dimorphism is expected to be different between species. On average, the protein-coding regions of the mouse and human genomes are 85 percent identical. Remaining genomic differences, plus non-coding regions, may impact the sex effect on metabolism in a different way in mice and humans. Thus, validation of sex disparities must be performed in translational studies from animal models to human. On average, the protein-coding regions of the mouse and human genomes are 85 percent identical. Some regions are highly evolutionarily conserved because they are required for function. In contrast, the non-coding regions are much less similar (only 50 percent or less). Since mice (or other animal models) have different life spans and maturational rates from humans, the findings from animal models may only serve as a reference for metabolism and pathophysiology in human diseases. The major impacts caused by gene homologue mutations may exceed the differences in sexual dimorphism between different species, as exemplified for genes Dhfr, Idh1, Mfap4, Nek2, Npc2, Phyh and Sra1.

We also found that the magnitude of the overall genotype effects on phenotypic traits and cellular metabolites was strikingly similar, with 26.3% of IMPC phenotypes (21.3% when excluding body weight measured at different time points, Supplementary Data S6) in KO lines differing from WT, and 21.5% of all metabolites being significantly affected by genotype effect or genotype–sex interactions. This finding supports the concept that cellular metabolites provide additional information on gene function. Of these overall gene KO effects, 37.4% of the phenotypes (30.7% when excluding body weight measured at different time points) and 34.4% of the metabolites were found to be sexually dimorphic. This proportion of differences between sexes in phenotypes and metabolites caused by KO mutations reinforces the importance of using both sexes when probing gene function. The differentiation of gene function by sex should be considered as an important factor in human disease etiology. Each of the 30 tested gene KO lines had clear and significant genotype or genotype–sex interaction effects on metabolism. This was true even for genes that had no direct impact on metabolic enzymes, such as C8a, a gene involved in immune response, or Dync1li1, a gene involved in intracellular protein transport and assembly. The mechanistic explanation for metabolomic sex differences remains to be investigated in detail. While we restricted analyses to plasma metabolite levels in order to study the potential translation of mouse screens for clinical use, it is clear that plasma levels serve only as indirect footprints of cellular, tissue and organ mechanisms underlying metabolic changes. Future studies using these and other gene KO lines will shed light on the target-specific effects of gene dysfunction and consequent impact on the metabolome. This, along with the metabolic data presented here, should in turn provide the basis for developing diagnostic tests for cell-to-organ system dysfunction and diseases.

Nevertheless, it was interesting that for several enzyme KOs, the effect of the perturbed biochemical reaction was directly detectable in plasma levels of the product metabolites. For example, IDH1 is one of three isoforms in mammals that catalyze the conversion of isocitrate to α-ketoglutarate. The activity of the other two isoforms could have had compensatory effects that might have led to undetectable changes on blood metabolites. Yet, both Idh1−/− male and female mice showed the clear upregulation of plasma isocitrate and the downregulation of plasma α-ketoglutarate with the same effect size. This initial perturbation of a major metabolic enzyme then caused additional downstream differences, specifically in lipid metabolites, such as reduced long-chain fatty acid, and increased mitochondrial acylcarnitine levels in both sexes. Yet, even for IDH1, important sexually dimorphic levels of plasma metabolites were observed, such as diametrically opposed changes in lyso-phosphatidylcholine and plasmanyl-phosphatidylcholine levels.

Previously Karp et al. analyzed 234 phenotype parameters from 2186 KO mouse lines across 10 IMPC centers and showed that phenotypic sexual dimorphism varied between centers [33]. For example, continuous phenotype trait parameters from TCP showed less sexual dimorphism than the average across all centers. The reason for this may be that the genes analyzed in one center inherently have less sexual dimorphism than the other centers. Indeed, sexually dimorphic phenotypes were higher in KO lines of genes expressing proteins that influence hormonal effects on behavior and physiology [33]. Several of the KO lines analyzed in this study included genes expressing proteins known to be involved in the regulation of sexual development (e.g., Sra1) [94]. The steroid receptor RNA activator protein SRAP, encoded by Sra1, regulates estrogen- and androgen-receptor-signaling pathways. Sra1 is an estrogen- and androgen-dependent gene that contributes to the progression of breast cancer in women [95]. Mitochondrial dysfunction and increased fatty acid oxidation were shown to be associated with breast cancer [96,97]. Twenty-three percent of all metabolites were affected in the Sra1−/− mice, and more than twice as many metabolites, including lipids were affected in Sra1−/− female mice compared to Sra1−/− male mice. In addition to lipids and acylcarnitines that showed sexual dimorphism in Sra1−/− female and male mice, several oxylipins, such as 11,12-EpETrE, 11-HETE and 15-HETE, were also differentially affected between the two sexes of Sra1−/− mice. Epoxyeicosatrienoic acids (EETs, or EpETrEs) induce angiogenesis and initiate cancer cell migration [98]. On the other hand, oxylipins, like 11-HETE are reported to have anti-mitogenic and anti-tumor activity [99,100]. Dysregulated plasma oxylipin levels in breast cancer patients indicated that these metabolites may become therapeutic target candidates [59,97,101], indicating that such gene–metabolite functional data may provide better understanding of the role(s) of a gene in metabolic regulation and its involvement in human diseases, including breast cancer. It also proved how data from mouse gene KOs could inform translational research into human diseases.

Several of the KO mouse lines analyzed have been shown previously to be associated with human disease phenotypes. For instance, mutations in human PHYH causes Refsum disease [102] which includes visual impairment and hearing loss. PHYH and its mouse orthologue, Phyh, encode a peroxisomal protein that is involved in the alpha-oxidation of 3-methyl branched fatty acids. Alpha-oxidation is a process in which fatty acids are shortened by one carbon atom, producing 2-hydroxylated intermediates in the process [102,103]. Our metabolomic results indicated the deletion of Phyh differentially affected peroxisomal alpha-oxidation-related metabolites of 2-hydroxylated (branched) fatty acids and their derivatives. Phyh−/− mutants also showed impacts on metabolites that are related to generic peroxisomal functions [103], including the downregulation of straight-chain fatty-acyl carnitine transport molecules. Other metabolites involved in peroxisomal oxidation were differentially altered, such as prostanoids (progesterone, PGD2 and PGF3alpha) and bile acids (tauroursodeoxycholic acid and glycocholic acid). Plasma metabolomic changes in Phyh−/− mice ranged from the differential regulation of methylated and acetylated amino acids to oxylipins, including 13-KODE; 15,16-DiHODE; 9,10-DiHODE; and 9,10-DiHOME, that were found in a sexually dimorphic manner (Supplementary Data S5). Therefore, our metabolic phenotype results confirm the primary role of Phyh but also adds valuable additional data to inform secondary mechanisms that link disease phenotypes to the underlying metabolic function(s). Another example is Npc2, which causes Niemann–Pick disease C2, a hereditary neurovisceral lysosomal lipid storage disorder due to mutations in NPC2. Respiratory distress and lung disease were uniformly observed in patients with Niemann–Pick type C2 in early infancy [62,104,105]. Mfap4-encoding microfibrillar-associated protein 4 (MFAP4) contributes to mature elastic fiber homeostasis and stability in connective tissues, such as lung, skin and aorta [106]. Plasma MFAP4 is associated with chronic obstructive pulmonary disease (COPD) severity and may serve as a COPD biomarker [107]. With a similar localization in tissues and implication in diseases of MFAP4 and NPC2, we were prompted to investigate to what extent they impact plasma metabolomics. Indeed, cholesterol and cholesterol esters showed similar alteration in plasma from Mfap4−/− and Npc2+/− mice. But they also had very different influences on most other metabolites with diverse genotype–sex interaction effects, indicating that genes involved in the same disease may exhibit effects on similar metabolic classes while affecting other metabolic phenotypes differently.

In addition, we found numerous interesting correlations between metabolomics and IMPC phenotypes. Correlation analysis (like Spearman rank calculations used in this study) rely on multiple data points to associate different variables. While the number of samples per KO genotype was too small to confidently score associations between IMPC phenotypes and plasma metabolites, the strength, number and the degree of sexual dimorphism of such correlations in female and male wildtype mice was astounding. While no one should mistake statistical associations with causal effects, we pose that associating visible IMPC phenotypes with molecular and metabolic variables may open the doors to better mechanistic understanding of mouse phenotypes, and eventually their links to corresponding human diseases. For example, center average speed is a common parameter used in mouse behavior and neurological and nerve system research. The blood triacylglyceride (16:0–18:0–22:0) consisting only of saturated fatty acyl groups showed completely opposite correlations with center average speed in wildtype female and male mice. The corresponding free palmitic acid was found to be involved in the sexual dimorphisms of microglia (resident brain immune cells) [108], while its hydroxylated fatty acid ester derivative, 5-PAHSA, was involved in antioxidant response in mice and PC12 neuronal model cells [108,109]. While few studies have investigated how specific triacylglycerides are related to disease phenotypes [110], these studies show possible ways to mechanistically relate lipids to neurological differences, at least in cell and animal models.

## 5. Conclusions

By adopting specific statistical methods [46,111] we demonstrated significant sexual dimorphism in both 30 KO lines and WT controls [112]. Sex must be considered as an important factor in interpreting the role(s) of a gene in metabolism and disease etiology. Such observations may serve to help understand the physiological consequences of genetic alterations underlying human diseases that manifest differently in men and women. In human diseases, sexual dimorphism is known for type 2 diabetes, cardiovascular disease, neurodegenerative diseases, autoimmunity, cancer, infectious diseases (such as COVID-19) and others [113,114,115]. Plasma metabolite alterations detected by metabolomics techniques can provide insight into and contribute to unraveling the many complex links between gene functions and the etiology of complex diseases. Sexual dimorphism of metabolites, together with other factors/omics, can be used to explain the underlying mechanism of the sex-specific progress of human diseases. With an overview of 30 mouse KO lines, we showed in this study how comprehensive metabolomics data may inform such links and how often metabolic effects are different between sexes. This report exemplifies the power for unraveling the links between gene functions and mouse models of human diseases once metabolomics data are combined with mouse phenotypes on the complement of >7000 available IMPC mouse KO lines.

## Figures and Tables

**Table 1:** Examples of top metabolites with sexual dimorphism in wildtype mice and their alterations in 30 knockout lines. Two-way ANOVA was used for statistical analysis. *, *p* < 0.05; **, *p* < 0.01, ***, *p* < 0.001.

	Metabolite	Sex Effect in WT	Statistical Analysis in Knockout Lines	
			Genotype Effect	Genotype—Sex Interaction
higher levels in female WT mice	Glycocholic acid	**	*Ahcy, C8a, Cdk4, Dhfr, Galc, Idh1, Lmbrd1, Mfap4, Mmachc, Mvk, Pebp1, Phyh, Ptpn12, Rock1, Ulk3, Ywhaz*	*Ahcy, C8a, Cdk4, Dhfr, Lmbrd1, Mfap4, Mvk, Phyh, Ptpn12, Rock1, Ulk3, Ywhaz*
	TMAO	***	*Ahcy, Atp5b, C8a, Dhfr, Dync1li1, Gnpda1, Lmbrd1, Npc2, Pebp1, Pipox, Plk1*	*Plk1*
	Tauroursodeoxycholic acid	*	*Ahcy, C8a, Cdk4, Dhfr, Dync1li1, G6pd2, Galc, Gnpda1, Idh1, Lmbrd1, Mmachc, Mvk, Nek2, Npc2, Pebp1, Phyh, Ptpn12, Pttg1, Rock1, Ulk3, Ywhaz*	*Ahcy, C8a, Dhfr, Lmbrd1, Mvk, Nek2, Npc2, Pttg1*
	Chenodeoxycholic acid	**	*Atp5b, Dhfr, Pmm2, Pttg1*	*/*
	Progesterone	**	*A2m, Atp6v0d1, Dync1li1, G6pd2, Galc, Mfap4, Phyh, Plk1, Pmm2, Sra1*	*A2m, Atp6v0d1, Plk1*
	2-Indolinone	***	*A2m, Ahcy, Atp5b, Atp6v0d1, C8a, Dhfr, Dync1li1, G6pd2, Galc, Gnpda1, Iqgap1, Lmbrd1, Mfap4, Mmachc, Mvk, Nek2, Npc2, Pebp1, Phyh, Plk1, Pmm2, Ptpn12, Pttg1, Rock1, Sra1, Ywhaz*	*/*
	PGF3alpha	**	*Lmbrd1, Mfap4, Mmachc, Pebp1, Phyh, Plk1, Ptpn12, Ywhaz*	*Lmbrd1, Mfap4, Pebp1, Phyh*
	11,12-EpETrE	***	*Atp5b, Mvk, Sra1*	*Mvk*
	SM d36:2	***	*Atp5b, Atp6v0d1, C8a, Lmbrd1, Pmm2, Ptpn12*	*C8a*
	HexCer-NS d18:1/16:0	***	*C8a, Sra1*	*C8a*
higher levels in male WT mice	Cer-NS d18:2/22:0	***	*C8a, Dync1li1, G6pd2, Pebp1, Pttg1, Sra1, Ulk3*	*Dync1li1, G6pd2, Pebp1, Sra1*
	PC 40:4	***	*/*	*/*
	PC 38:2	***	*A2m, Atp5b, Dync1li1, Pebp1, Pmm2, Pttg1, Sra1*	*Sra1*
	Testosterone	***	*Atp5b, Plk1*	*/*
	LPC 20:0	***	*A2m, Cdk4, Dhfr, Dync1li1, Iqgap1, Pebp1, Sra1, Ulk3*	*/*
	PI 16:0–16:1	***	*A2m, Galc, Idh1, Nek2, Pebp1, Sra1, Ywhaz*	*Galc, Idh1, Nek2, Pebp1*
	PE 18:0–22:5	***	*Ahcy, Galc, Pttg1*	*Ahcy*
	Cer (d18:1(4E)/22:0)	***	*Dync1li1, G6pd2, Pebp1, Sra1, G6pd2*	*Sra1*
	PC 36:6	***	*Gnpda1, Phyh, Ptpn12, Ulk3*	*Ptpn12*
	TAG 14:0–16:0–18:2	***	*A2m, Dync1li1, Lmbrd1, Npc2, Pebp1*	*Npc2, Pebp1*

## Data Availability

R code for reproducing the analyses shown in the main figures is available at https://github.com/ythzhang/KOMP_test, accessed on 3 August 2023. Raw and processed data are available at the NIH Metabolomics Workbench database (http://metabolomicsworkbench.org, accessed on 3 August 2023) (Accession number ST001154).

## Supplementary Materials

The following supporting information can be downloaded at: https://www.mdpi.com/article/10.3390/metabo13080947/s1, Figure S1: Statistical analysis designs to study sexual dimorphism in wildtype mice and in knockout mice; Figure S2: Sex as a biological variable in metabolomics data with a stricter significance threshold and adjusted to body mass in wildtype C57BL/6NCrl mice. Figure S3: Sexual dimorphism of metabolomics data and phenotype data in wildtype mice; Figure S4: PLS-DA plots of metabolomic data in 30 KO mouse lines compared to wildtype mice; Figure S5: Heatmaps of ChemRICH set enrichment clusters for female (F) and male (M) wildtype mice, calculated from Spearman rank correlations of metabolite versus IMPC phenotypes. Table S1: Gene information of 30 knockout lines; Table S2: Number of significantly changed metabolites by genotype effect and their alterations in 30 knockout strains; Table S3: Examples of sexual dimorphism of phenotypes (continuous traits) in wildtype mice and their alterations in 30 knockout lines. Supplementary Data S1: phenotype data; Supplementary Data S2: metabolite data; Supplementary Data S3: WT metabolite differences; Supplementary Data S4: WT phenotype statistics; Supplementary Data S5: Knockout 2-way ANOVA (data for Figure 3); Supplementary Data S6: Knockout gene-sex interactions; Supplementary Data S7: Metabolite-phenotype associations.

## References

[1] Mauvais-Jarvis F., Arnold A.P., Reue K. (2017). A Guide for the Design of Pre-clinical Studies on Sex Differences in Metabolism. Cell Metab..

[2] Tam A., Churg A., Wright J.L., Zhou S., Kirby M., Coxson H.O., Lam S., Man S.F.P., Sin D.D. (2016). Sex Differences in Airway Remodeling in a Mouse Model of Chronic Obstructive Pulmonary Disease. Am. J. Respir. Crit. Care Med..

[3] Hanamsagar R., Alter M.D., Block C.S., Sullivan H., Bolton J.L., Bilbo S.D. (2017). Generation of a microglial developmental index in mice and in humans reveals a sex difference in maturation and immune reactivity. Glia.

[4] Mauvais-Jarvis F. (2015). Sex differences in metabolic homeostasis, diabetes, and obesity. Biol. Sex Differ..

[5] Arnold A.P., Cassis L.A., Eghbali M., Reue K., Sandberg K. (2017). Sex Hormones and Sex Chromosomes Cause Sex Differences in the Development of Cardiovascular Diseases. Arterioscler. Thromb. Vasc. Biol..

[6] Ratnu V.S., Emami M.R., Bredy T.W. (2017). Genetic and epigenetic factors underlying sex differences in the regulation of gene expression in the brain. J. Neurosci. Res..

[7] Klein S.L., Flanagan K.L. (2016). Sex differences in immune responses. Nat. Rev. Immunol..

[8] Regitz-Zagrosek V., Kararigas G. (2017). Mechanistic Pathways of Sex Differences in Cardiovascular Disease. Physiol. Rev..

[9] Labonté B., Engmann O., Purushothaman I., Menard C., Wang J., Tan C., Scarpa J.R., Moy G., E Loh Y.-H., Cahill M. (2017). Sex-specific transcriptional signatures in human depression. Nat. Med..

[10] McCarthy M.M., Nugent B.M. (2015). At the frontier of epigenetics of brain sex differences. Front. Behav. Neurosci..

[11] Pollitzer E. (2013). Biology: Cell sex matters. Nature.

[12] Clayton J.A., Collins F.S. (2014). Policy: NIH to balance sex in cell and animal studies. Nature.

[13] Zucker I., Beery A.K. (2010). Males still dominate animal studies. Nature.

[14] Palanza P., Parmigiani S. (2017). How does sex matter? Behavior, stress and animal models of neurobehavioral disorders. Neurosci. Biobehav. Rev..

[15] Florez-Vargas O., Brass A., Karystianis G., Bramhall M., Stevens R., Cruickshank S., Nenadic G. (2016). Bias in the reporting of sex and age in biomedical research on mouse models. Elife.

[16] Beery A.K., Zucker I. (2011). Sex bias in neuroscience and biomedical research. Neurosci. Biobehav. Rev..

[17] National Institutes of Health NIH Policy on Sex as a Biological Variable. https://orwh.od.nih.gov/sex-gender/nih-policy-sex-biological-variable.

[18] Quinn M., Ramamoorthy S., Cidlowski J.A. (2014). Sexually dimorphic actions of glucocorticoids: Beyond chromosomes and sex hormones. Ann. N. Y. Acad. Sci..

[19] Lauretta R., Sansone M., Sansone A., Romanelli F., Appetecchia M. (2018). Gender in Endocrine Diseases: Role of Sex Gonadal Hormones. Int. J. Endocrinol..

[20] Imtiaz B., Tuppurainen M., Rikkonen T., Kivipelto M., Soininen H., Kröger H., Tolppanen A.M. (2017). Postmenopausal hormone therapy and Alzheimer disease: A prospective cohort study. Neurology.

[21] Jabbar A., Pingitore A., Pearce S.H.S., Zaman A., Iervasi G., Razvi S. (2017). Thyroid hormones and cardiovascular disease. Nat. Rev. Cardiol..

[22] Nauck M.A., Meier J.J. (2018). Incretin hormones: Their role in health and disease. Diabetes Obes. Metab..

[23] Gribble F.M., Reimann F. (2019). Function and mechanisms of enteroendocrine cells and gut hormones in metabolism. Nat. Rev. Endocrinol..

[24] Audano M., Maldini M., De Fabiani E., Mitro N., Caruso D. (2018). Gender-related metabolomics and lipidomics: From experimental animal models to clinical evidence. J. Proteom..

[25] Stanley E.G., Bailey NJ C., Bollard M.E., Haselden J.N., Waterfield C.J., Holmes E., Nicholson J.K. (2005). Sexual dimorphism in urinary metabolite profiles of Han Wistar rats revealed by nuclear-magnetic-resonance-based metabonomics. Anal. Biochem..

[26] Honma A., Revell V.L., Gunn P.J., Davies S.K., Middleton B., Raynaud F.I., Skene D.J. (2020). Effect of acute total sleep deprivation on plasma melatonin, cortisol and metabolite rhythms in females. Eur. J. Neurosci..

[27] Ellul S., Ponsonby A.-L., Carlin J.B., Collier F., Mansell T., Vuillermin P., Burgner D., Saffery R., Barwon Infant Study Investigator Team (2020). Sex differences in infant blood metabolite profile in association with weight and adiposity measures. Pediatr. Res..

[28] Wells J.C. (2007). Sexual dimorphism of body composition. Best Pract. Res. Clin. Endocrinol. Metab..

[29] Janssen I., Heymsfield S.B., Wang Z.M., Ross R. (2000). Skeletal muscle mass and distribution in 468 men and women aged 18–88 yr. J. Appl. Physiol..

[30] Greenman A.C., Albrecht D.M., Halberg R.B., Diffee G.M. (2020). Sex differences in skeletal muscle alterations in a model of colorectal cancer. Physiol. Rep..

[31] Karastergiou K., Smith S.R., Greenberg A.S., Fried S.K. (2012). Sex differences in human adipose tissues—The biology of pear shape. Biol. Sex Differ..

[32] Ring N., Meehan T.F., Blake A., Brown J., Chen C.-K., Conte N., Di Fenza A., Fiegel T., Horner N., Jacobsen J.O.B. (2015). A mouse informatics platform for phenotypic and translational discovery. Mamm. Genome.

[33] Karp N.A., Mason J., Beaudet A.L., Benjamini Y., Bower L., Braun R.E., Brown S.D., Chesler E.J., Dickinson M.E., Flenniken A.M. (2017). Prevalence of sexual dimorphism in mammalian phenotypic traits. Nat. Commun..

[34] Dunn W.B., Broadhurst D.I., Atherton H.J., Goodacre R., Griffin J.L. (2011). Systems level studies of mammalian metabolomes: The roles of mass spectrometry and nuclear magnetic resonance spectroscopy. Chem. Soc. Rev..

[35] Pinu F.R., Beale D.J., Paten A.M., Kouremenos K., Swarup S., Schirra H.J., Wishart D. (2019). Systems Biology and Multi-Omics Integration: Viewpoints from the Metabolomics Research Community. Metabolites.

[36] Menni C., Zierer J., Valdes A.M., Spector T.D. (2017). Mixing omics: Combining genetics and metabolomics to study rheumatic diseases. Nat. Rev. Rheumatol..

[37] Johnson C.H., Ivanisevic J., Siuzdak G. (2016). Metabolomics: Beyond biomarkers and towards mechanisms. Nat. Rev. Mol. Cell Biol..

[38] Cajka T., Fiehn O. (2016). Toward Merging Untargeted and Targeted Methods in Mass Spectrometry-Based Metabolomics and Lipidomics. Anal. Chem..

[39] Fiehn O. (2016). Metabolomics by Gas Chromatography-Mass Spectrometry: Combined Targeted and Untargeted Profiling. Curr. Protoc. Mol. Biol..

[40] Wishart D.S., Feunang Y.D., Marcu A., Guo A.C., Liang K., Vázquez-Fresno R., Sajed T., Johnson D., Li C., Karu N. (2018). HMDB 4.0: The human metabolome database for 2018. Nucleic Acids Res..

[41] Siskos A.P., Jain P., Römisch-Margl W., Bennett M., Achaintre D., Asad Y., Marney L., Richardson L., Koulman A., Griffin J.L. (2017). Interlaboratory Reproducibility of a Targeted Metabolomics Platform for Analysis of Human Serum and Plasma. Anal. Chem..

[42] Miller M.J., Kennedy A.D., Eckhart A.D., Burrage L.C., Wulff J.E., Miller L.A., Milburn M.V., Ryals J.A., Beaudet A.L., Sun Q. (2015). Untargeted metabolomic analysis for the clinical screening of inborn errors of metabolism. J. Inherit. Metab. Dis..

[43] Kennedy A.D., Wittmann B.M., Evans A.M., Miller L.A., Toal D.R., Lonergan S., Elsea S.H., Pappan K.L. (2018). Metabolomics in the clinic: A review of the shared and unique features of untargeted metabolomics for clinical research and clinical testing. J. Mass. Spectrom..

[44] Barupal D.K., Zhang Y., Shen T., Fan S., Roberts B.S., Fitzgerald P., Wancewicz B., Valdiviez L., Wohlgemuth G., Byram G. (2019). A Comprehensive Plasma Metabolomics Dataset for a Cohort of Mouse Knockouts within the International Mouse Phenotyping Consortium. Metabolites.

[45] Koscielny G., Yaikhom G., Iyer V., Meehan T.F., Morgan H., Atienza-Herrero J., Blake A., Chen C.K., Easty R., Di Fenza A. (2014). The International Mouse Phenotyping Consortium Web Portal, a unified point of access for knockout mice and related phenotyping data. Nucleic Acids Res..

[46] Barupal D.K., Fiehn O. (2017). Chemical Similarity Enrichment Analysis (ChemRICH) as alternative to biochemical pathway mapping for metabolomic datasets. Sci. Rep..

[47] Moore B.A., Flenniken A.M., Clary D., Moshiri A.S., Nutter L.M., Berberovic Z., Owen C., Newbigging S., Adissu H., Eskandarian M. (2019). Genome-wide screening of mouse knockouts reveals novel genes required for normal integumentary and oculocutaneous structure and function. Sci. Rep..

[48] Wang T.J., Larson M.G., Vasan R.S., Cheng S., Rhee E.P., McCabe E., Lewis G.D., Fox C.S., Jacques P.F., Fernandez C. (2011). Metabolite profiles and the risk of developing diabetes. Nat. Med..

[49] Mittelstrass K., Ried J.S., Yu Z., Krumsiek J., Gieger C., Prehn C., Roemisch-Margl W., Polonikov A., Peters A., Theis F.J. (2011). Discovery of sexual dimorphisms in metabolic and genetic biomarkers. PLoS Genet..

[50] Krumsiek J., Mittelstrass K., Do K.T., Stückler F., Ried J., Adamski J., Peters A., Illig T., Kronenberg F., Friedrich N. (2015). Gender-specific pathway differences in the human serum metabolome. Metabolomics.

[51] van der Molen H.J., Groen D. (1965). Determination of progesterone in human peripheral blood using gas-liquid chromatography with electron capture detection. J. Clin. Endocrinol. Metab..

[52] Barrea L., Annunziata G., Muscogiuri G., Laudisio D., Di Somma C., Maisto M., Tenore G.C., Colao A., Savastano S. (2019). Trimethylamine N-oxide, Mediterranean diet, and nutrition in healthy, normal-weight adults: Also a matter of sex?. Nutrition.

[53] Rushworth D., Mathews A., Alpert A., Cooper L.J.N. (2015). Dihydrofolate Reductase and Thymidylate Synthase Transgenes Resistant to Methotrexate Interact to Permit Novel Transgene Regulation. J. Biol. Chem..

[54] Fry A.M. (2002). The Nek2 protein kinase: A novel regulator of centrosome structure. Oncogene.

[55] Mlodzik M. (2016). The Dishevelled Protein Family: Still Rather a Mystery After Over 20 Years of Molecular Studies. Curr. Top Dev. Biol..

[56] Hoglinger D., Burgoyne T., Sanchez-Heras E., Hartwig P., Colaco A., Newton J., Futter C.E., Spiegel S., Platt F.M., Eden E.R. (2019). NPC1 regulates ER contacts with endocytic organelles to mediate cholesterol egress. Nat. Commun..

[57] Robinson J.I., Weir W.H., Crowley J.R., Hink T., Reske K.A., Kwon J.H., Burnham C.A., Dubberke E.R., Mucha P.J., Henderson J.P. (2019). Metabolomic networks connect host-microbiome processes to human Clostridioides difficile infections. J. Clin. Investig..

[58] Lytra G., Miot-Sertier C., Moine V., Coulon J., Barbe J.-C. (2020). Influence of must yeast-assimilable nitrogen content on fruity aroma variation during malolactic fermentation in red wine. Food Res. Int..

[59] Chocholouskova M., Jirásko R., Vrána D., Gatěk J., Melichar B., Holčapek M. (2019). Reversed phase UHPLC/ESI-MS determination of oxylipins in human plasma: A case study of female breast cancer. Anal. Bioanal. Chem..

[60] Zou Z., Bellenger S., Massey K.A., Nicolaou A., Geissler A., Bidu C., Bonnotte B., Pierre A.S., Minville-Walz M., Rialland M. (2013). Inhibition of the HER2 pathway by n-3 polyunsaturated fatty acids prevents breast cancer in fat-1 transgenic mice. J. Lipid Res..

[61] Fagerberg L., Hallström B.M., Oksvold P., Kampf C., Djureinovic D., Odeberg J., Habuka M., Tahmasebpoor S., Danielsson A., Edlund K. (2014). Analysis of the human tissue-specific expression by genome-wide integration of transcriptomics and antibody-based proteomics. Mol. Cell. Proteom..

[62] Guillemot N., Troadec C., de Villemeur T.B., Clement A., Fauroux B. (2007). Lung disease in Niemann-Pick disease. Pediatr. Pulmonol..

[63] Brandsma C.A., van den Berge M., Postma D.S., Jonker M.R., Brouwer S., Paré P.D., Sin D.D., Bossé Y., Laviolette M., Karjalainen J. (2015). A large lung gene expression study identifying fibulin-5 as a novel player in tissue repair in COPD. Thorax.

[64] Jafari Z., Kolb B.E., Mohajerani M.H. (2020). Prepulse inhibition of the acoustic startle reflex and P50 gating in aging and alzheimer’s disease. Ageing Res. Rev..

[65] Djoumbou Feunang Y., Eisner R., Knox C., Chepelev L., Hastings J., Owen G., Fahy E., Steinbeck C., Subramanian S., Bolton E. (2016). ClassyFire: Automated chemical classification with a comprehensive, computable taxonomy. J. Cheminformatics.

[66] Coll A.P., Farooqi I.S., O’Rahilly S. (2007). The hormonal control of food intake. Cell.

[67] O’Shaughnessy P.J. (2014). Hormonal control of germ cell development and spermatogenesis. Semin. Cell Dev. Biol..

[68] Taniguchi M., Okazaki T. (2014). The role of sphingomyelin and sphingomyelin synthases in cell death, proliferation and migration-from cell and animal models to human disorders. Biochim. Biophys. Acta.

[69] Nayeem M.A. (2018). Role of oxylipins in cardiovascular diseases. Acta Pharmacol. Sin..

[70] Alexeev E.E., Lanis J.M., Kao D.J., Campbell E.L., Kelly C.J., Battista K.D., Gerich M.E., Jenkins B.R., Walk S.T., Kominsky D.J. (2018). Microbiota-Derived Indole Metabolites Promote Human and Murine Intestinal Homeostasis through Regulation of Interleukin-10 Receptor. Am. J. Pathol..

[71] Swann J.R., Want E.J., Geier F.M., Spagou K., Wilson I.D., Sidaway J.E., Nicholson J.K., Holmes E. (2011). Systemic gut microbial modulation of bile acid metabolism in host tissue compartments. Proc. Natl. Acad. Sci. USA.

[72] Wang Z., Klipfell E., Bennett B.J., Koeth R., Levison B.S., DuGar B., Feldstein A.E., Britt E.B., Fu X., Chung Y.M. (2011). Gut flora metabolism of phosphatidylcholine promotes cardiovascular disease. Nature.

[73] Johnson C., Prokopienko A.J., West R.E., Nolin T.D., Stubbs J.R. (2018). Decreased Kidney Function Is Associated with Enhanced Hepatic Flavin Monooxygenase Activity and Increased Circulating Trimethylamine N-Oxide Concentrations in Mice. Drug Metab. Dispos..

[74] Cho C.E., Caudill M.A. (2017). Trimethylamine-N-Oxide: Friend, Foe, or Simply Caught in the Cross-Fire?. Trends Endocrinol. Metab..

[75] Tang W.H., Wang Z., Levison B.S., Koeth R.A., Britt E.B., Fu X., Wu Y., Hazen S.L. (2013). Intestinal microbial metabolism of phosphatidylcholine and cardiovascular risk. N. Engl. J. Med..

[76] Kim R.B., Morse B.L., Djurdjev O., Tang M., Muirhead N., Barrett B., Holmes D.T., Madore F., Clase C.M., Rigatto C. (2016). Advanced chronic kidney disease populations have elevated trimethylamine N-oxide levels associated with increased cardiovascular events. Kidney Int..

[77] Manor O., Zubair N., Conomos M.P., Xu X., Rohwer J.E., Krafft C.E., Lovejoy J.C., Magis A.T. (2018). A Multi-omic Association Study of Trimethylamine N-Oxide. Cell Rep..

[78] Organ C.L., Otsuka H., Bhushan S., Wang Z., Bradley J., Trivedi R., Polhemus D.J., Tang W.W., Wu Y., Hazen S.L. (2016). Choline Diet and Its Gut Microbe-Derived Metabolite, Trimethylamine N-Oxide, Exacerbate Pressure Overload-Induced Heart Failure. Circ. Heart Fail..

[79] Papandreou C., Bulló M., Zheng Y., Ruiz-Canela M., Yu E., Guasch-Ferré M., Toledo E., Clish C., Corella D., Estruch R. (2018). Plasma trimethylamine-N-oxide and related metabolites are associated with type 2 diabetes risk in the Prevencion con Dieta Mediterranea (PREDIMED) trial. Am. J. Clin. Nutr..

[80] Mondul A.M., Moore S.C., Weinstein S.J., Karoly E.D., Sampson J.N., Albanes D. (2015). Metabolomic analysis of prostate cancer risk in a prospective cohort: The alpha-tocolpherol, beta-carotene cancer prevention (ATBC) study. Int. J. Cancer.

[81] Guertin K.A., Li X.S., Graubard B.I., Albanes D., Weinstein S.J., Goedert J.J., Wang Z., Hazen S.L., Sinha R. (2017). Serum Trimethylamine N-oxide, Carnitine, Choline, and Betaine in Relation to Colorectal Cancer Risk in the Alpha Tocopherol, Beta Carotene Cancer Prevention Study. Cancer Epidemiol. Biomark. Prev..

[82] Caligiuri S.P.B., Parikh M., Stamenkovic A., Pierce G.N., Aukema H.M. (2017). Dietary modulation of oxylipins in cardiovascular disease and aging. Am. J. Physiol. Heart Circ. Physiol..

[83] Imig J.D., Zhao X., Zaharis C.Z., Olearczyk J.J., Pollock D.M., Newman J.W., Kim I.H., Watanabe T., Hammock B.D. (2005). An orally active epoxide hydrolase inhibitor lowers blood pressure and provides renal protection in salt-sensitive hypertension. Hypertension.

[84] Jenkins C.M., Cedars A., Gross R.W. (2009). Eicosanoid signalling pathways in the heart. Cardiovasc. Res..

[85] Caligiuri S.P., Rodriguez-Leyva D., Aukema H.M., Ravandi A., Weighell W., Guzman R., Pierce G.N. (2016). Dietary Flaxseed Reduces Central Aortic Blood Pressure Without Cardiac Involvement but Through Changes in Plasma Oxylipins. Hypertension.

[86] Kander M.C., Cui Y., Liu Z. (2017). Gender difference in oxidative stress: A new look at the mechanisms for cardiovascular diseases. J. Cell. Mol. Med..

[87] Haemmerle G., Lass A. (2019). Genetically modified mouse models to study hepatic neutral lipid mobilization. Biochim. Biophys. Acta Mol. Basis Dis..

[88] Yang W., Xiao J., Yang Z., Ji L., Jia W., Weng J., Lu J., Shan Z., Liu J., Tian H. (2012). Serum lipids and lipoproteins in Chinese men and women. Circulation.

[89] Wang X., Magkos F., Mittendorfer B. (2011). Sex differences in lipid and lipoprotein metabolism: It’s not just about sex hormones. J. Clin. Endocrinol. Metab..

[90] Palmisano B.T., Zhu L., Eckel R.H., Stafford J.M. (2018). Sex differences in lipid and lipoprotein metabolism. Mol. Metab..

[91] Barupal D.K., Zhang Y., Fan S., Hazen S.L., Tang W.H., Cajka T., Irvin M.R., Arnett D.K., Kind T., Kaddurah-Daouk R. (2019). The circulating lipidome is largely defined by sex descriptors in the GOLDN, GeneBank and the ADNI studies. bioRxiv.

[92] Rist M.J., Roth A., Frommherz L., Weinert C.H., Krüger R., Merz B., Bunzel D., Mack C., Egert B., Bub A. (2017). Metabolite patterns predicting sex and age in participants of the Karlsruhe Metabolomics and Nutrition (KarMeN) study. PLoS ONE.

[93] Ellul S., Wake M., Clifford S.A., Lange K., Würtz P., Juonala M., Dwyer T., Carlin J.B., Burgner D.P., Saffery R. (2019). Metabolomics: Population epidemiology and concordance in Australian children aged 11–12 years and their parents. BMJ Open.

[94] Colley S.M., Leedman P.J. (2011). Steroid Receptor RNA Activator—A nuclear receptor coregulator with multiple partners: Insights and challenges. Biochimie.

[95] Cooper C., Vincett D., Yan Y., Hamedani M.K., Myal Y., Leygue E. (2011). Steroid Receptor RNA Activator bi-faceted genetic system: Heads or Tails?. Biochimie.

[96] Martinez-Outschoorn U.E., Sotgia F., Lisanti M.P. (2012). Power surge: Supporting cells “fuel” cancer cell mitochondria. Cell. Metab..

[97] Camarda R., Zhou A.Y., Kohnz R.A., Balakrishnan S., Mahieu C., Anderton B., Eyob H., Kajimura S., Tward A., Krings G. (2016). Inhibition of fatty acid oxidation as a therapy for MYC-overexpressing triple-negative breast cancer. Nat. Med..

[98] Apaya M.K., Chang M.-T., Shyur L.-F. (2016). Phytomedicine polypharmacology: Cancer therapy through modulating the tumor microenvironment and oxylipin dynamics. Pharmacol. Ther..

[99] Schweiger D., Furstenberger G., Krieg P. (2007). Inducible expression of 15-lipoxygenase-2 and 8-lipoxygenase inhibits cell growth via common signaling pathways. J. Lipid Res..

[100] Greene E.R., Huang S., Serhan C.N., Panigrahy D. (2011). Regulation of inflammation in cancer by eicosanoids. Prostaglandins. Other Lipid Mediat..

[101] Pakiet A., Kobiela J., Stepnowski P., Sledzinski T., Mika A. (2019). Changes in lipids composition and metabolism in colorectal cancer: A review. Lipids Health Dis..

[102] Wanders R.J., Waterham H.R. (2006). Peroxisomal disorders: The single peroxisomal enzyme deficiencies. Biochim. Biophys. Acta.

[103] Waterham H.R., Ferdinandusse S., Wanders R.J. (2016). Human disorders of peroxisome metabolism and biogenesis. Biochim. Biophys. Acta.

[104] Griese M., Brasch F., Aldana V.R., Cabrera M.M., Goelnitz U., Ikonen E., Karam B.J., Liebisch G., Linder M.D., Lohse P. (2010). Respiratory disease in Niemann-Pick type C2 is caused by pulmonary alveolar proteinosis. Clin. Genet..

[105] Bjurulf B., Spetalen S., Erichsen A., Vanier M.T., Strøm E.H., Strømme P. (2008). Niemann-Pick disease type C2 presenting as fatal pulmonary alveolar lipoproteinosis: Morphological findings in lung and nervous tissue. Med. Sci. Monit..

[106] Pilecki B., Holm A.T., Schlosser A., Moeller J.B., Wohl A.P., Zuk A.V., Heumüller S.E., Wallis R., Moestrup S.K., Sengle G. (2016). Characterization of Microfibrillar-associated Protein 4 (MFAP4) as a Tropoelastin- and Fibrillin-binding Protein Involved in Elastic Fiber Formation. J. Biol. Chem..

[107] Johansson S.L., Roberts N.B., Schlosser A., Andersen C.B., Carlsen J., Wulf-Johansson H., Sækmose S.G., Titlestad I.L., Tornoe I., Miller B. (2014). Microfibrillar-associated protein 4: A potential biomarker of chronic obstructive pulmonary disease. Respir. Med..

[108] Wang J.T., Yu Z.Y., Tao Y.H., Liu Y.C., Wang Y.M., Guo Q.L., Xue J.Z., Wen X.H., Zhang Q., Xu X.D. (2021). A novel palmitic acid hydroxy stearic acid (5-PAHSA) plays a neuroprotective role by inhibiting phosphorylation of the m-TOR-ULK1 pathway and regulating autophagy. CNS Neurosci. Ther..

[109] Yanguas-Casas N., Crespo-Castrillo A., de Ceballos M.L., Chowen J.A., Azcoitia I., Arevalo M.A., Garcia-Segura L.M. (2018). Sex differences in the phagocytic and migratory activity of microglia and their impairment by palmitic acid. Glia.

[110] Kwon H.-M., Lim J.-S., Park H.-K., Lee Y.-S. (2011). Hypertriglyceridemia as a possible predictor of early neurological deterioration in acute lacunar stroke. J. Neurol. Sci..

[111] Karp N.A., Heller R., Yaacoby S., White J.K., Benjamini Y. (2017). Improving the Identification of Phenotypic Abnormalities and Sexual Dimorphism in Mice When Studying Rare Event Categorical Characteristics. Genetics.

[112] Gertsenstein M., Nutter L.M., Reid T., Pereira M., Stanford W.L., Rossant J., Nagy A. (2010). Efficient generation of germ line transmitting chimeras from C57BL/6N ES cells by aggregation with outbred host embryos. PLoS ONE.

[113] Dai H., Zhang X., Zhang W., Wang Z., Qiu M. (2022). Editorial: The Role of Sex Dimorphism in Disease Susceptibility and Immune Response. Front. Nutr..

[114] Gay L., Melenotte C., Lakbar I., Mezouar S., Devaux C., Raoult D., Bendiane M.K., Leone M., Mège J.L. (2021). Sexual Dimorphism and Gender in Infectious Diseases. Front. Immunol..

[115] Martin-Grau M., Monleon D. (2023). Sex dimorphism and metabolic profiles in management of metabolic-associated fatty liver disease. World J. Clin. Cases.

