# Supplementary material for: Sexual Dimorphism of the Mouse Plasma Metabolome Is Associated with Phenotypes of 30 Gene Knockout Lines
Source: Metabolites. 2023 Aug 15;13(8):947. doi: 10.3390/metabo13080947 (PMC10456929; doi:10.3390/metabo13080947)
Supplement: Supplementary file 1 [file metabolites-13-00947-s001.zip › metabolites-2533412-supplementary.pdf]

Supplementary Information for

Sexual dimorphism of the mouse plasma metabolome is associated with phenotypes of 30 gene knockout lines

Ying Zhang[1,2], Dinesh K. Barupal[3], Sili Fan[1], Bei Gao[4], Chao Zhu[5], Ann M. Flenniken[6,7], Colin McKerlie[6,8], Lauryl M. J. Nutter[6,8], K. C. Kent Lloyd[9] and Oliver Fiehn[1*]

1 West Coast Metabolomics Center, University of California, Davis, Davis, CA 95616, United States
2 Department of Chemistry, University of California, Davis, Davis, CA 95616, United States
3 Department of Environmental Medicine and Public Health, Icahn School of Medicine at Mount Sinai, New York, NY 10029, United States; dinesh.barupal@mssm.edu
4 School of Marine Sciences, Nanjing University of Information Science and Technology, Nanjing, 210044, China
5 College of Medicine & Nursing, Dezhou University, De Zhou, Shandong 253023, China
6 The Centre for Phenogenomics, Toronto, ON M5T 3H7, Canada; flenniken@lunenfeld.ca (A.M.F.); colin.mckerlie@sickkids.ca (C.M.); lauryl.nutter@sickkids.ca (L.M.J.N.)
7 Lunenfeld-Tanenbaum Research Institute, Mount Sinai Hospital, Toronto, ON M5G 1X5, Canada
8 The Hospital for Sick Children, Toronto, ON M5G 1X8, Canada
9 Department of Surgery, School of Medicine, and Mouse Biology Program, University of California, Davis, Davis, CA 95616, United States; kclloyd@ucdavis.edu
* Correspondence: ofiehn@ucdavis.edu

Supplementary Tables:

Supplementary Table S1. Gene information of 30 knockout lines

Supplementary Table S2. Number of significantly changed metabolites by genotype effect and their alterations in 30 knockout strains.

Supplementary Table S3. Examples of sexual dimorphism of phenotypes (continuous traits) in wildtype mice and their alterations in 30 knockout lines.

Supplementary Figures:

Supplementary Figure S1. Statistical analysis designs to study sexual dimorphism in wildtype mice and in knockout mice.

Supplementary Figure S2. Sex as a biological variable in metabolomics data with a stricter significance threshold and adjusted to body mass in wildtype C57BL/6NCrl mice.

Supplementary Figure S3. Sexual dimorphism of metabolomics data and phenotype data in wildtype mice

Supplementary Figure S4. PLS-DA plots of metabolomic data in 30 KO mouse lines compared to wildtype mice

Supplementary Figure S5. Heatmaps of ChemRICH set enrichment clusters for female (F) and male (M) wildtype mice, calculated from Spearman rank correlations of metabolite versus IMPC phenotypes.

Online Datasets:

Supplementary Data S1: phenotype data
Supplementary Data S2: metabolite data
Supplementary Data S3: WT metabolite differences
Supplementary Data S4: WT phenotype statistics
Supplementary Data S5: Knockout 2-way ANOVA (data for Figure 3)
Supplementary Data S6: Knockout gene-sex interactions
Supplementary Data S7: Metabolite-phenotype associations

Supplementary TableS1. Gene information of 30 knockout lines							
Gene symbol	Name	Zygosity	Male		Female		Comment
			Age (wks)	Number	Age (wks)	Number	
None (wildtype control)	Null	+/+	16.1 ± 0.3	20	16.1 ± 0.3	20	/
A2m	Alpha-2-macroglobulin	-/-	15.7 ± 0	3	15.7 ± 0	3	Target protein assay
Ahcy	S-adenosylhomocysteine hydrolase	+/-	16.1 ± 0.2	3	16.2 ± 0.3	3	Enzyme on pathway reactions
Atp5b	ATP synthase, H+ transporting mitochondrial F1 complex, beta subunit	+/-	16.3 ± 0	3	16 ± 0.5	3	Target protein assay
Atp6v0d1	ATPase H+ Transporting lysosomal V0 Subunit D1	+/-	16.2 ± 0.1	3	15.9 ± 0	3	Random selection
C8a	Complement Componet 8, alpha polypeptide	-/-	16 ± 0.2	3	16.2 ± 0.6	3	Target protein assay
Cdk4	Cyclin Dependent Kinase 4	+/-	15.9 ± 0	3	16 ± 0.4	3	Random selection
Dhfr	Dihydrofolate reductase	+/-	16.3 ± 0	3	16.1 ± 0.4	3	Enzyme on pathway reactions
Dync1li1	Dynein Cytoplasmic 1 Light Intermediate Chain 1	+/-	16 ± 0.3	3	16.4 ± 0	3	Random selection
G6pd2	Glucose 6-phosphate dehydrogenase 2	-/-	16.4 ± 0.3	3	15.9 ± 0.1	3	Enzyme on pathway reactions
Galc	Galactosylceramidase	+/-	16.1 ± 0	3	16 ± 0	3	Enzyme on pathway reactions
Gnpda1	Glucosamine-6-phosphate deaminase 1	+/-	16.3 ± 0.1	3	16.3 ± 0.1	3	Enzyme on pathway reactions
Idh1	Isocitrate dehydrogenase	-/-	16 ± 0.2	3	16.1 ± 0.4	3	Random selection
Iqgap1	IQ motif containing GTPase activating protein 1	-/-	15.8 ± 0.1	3	15.9 ± 0.1	3	Random selection
Lmbrd1	LMBR1 domain containing 1	+/-	16 ± 0.2	3	15.9 ± 0.2	3	Enzyme on pathway reactions
Mfap4	Microfibrillar-associated protein 4	-/-	16 ± 0.2	3	16.6 ± 0	3	Target protein assay
Mmachc	Methylmalonic aciduria cblC type, with homocystinuria	+/-	16 ± 0.4	3	16 ± 0.2	3	Enzyme on pathway reactions
Mvk	mevalonate kinase	+/-	16.4 ± 0.1	3	15.7 ± 0.1	3	Random selection
Nek2	NIMA (never in mitosis gene a)-related expressed kinase 2	-/-	15.8 ± 0.2	3	16 ± 0.1	3	Random selection
Npc2	NPC intracellular cholesterol transporter 2	+/-	16 ± 0.2	3	16.2 ± 0.2	3	Enzyme on pathway reactions
Pebp1	Phosphatidylethanolamine binding protein 1	-/-	16.3 ± 0.2	3	16 ± 0.1	3	Target protein assay
Phyh	Phytanoyl- CoA hydroxylase	-/-	16.1 ± 0.3	3	16 ± 0.3	3	Random selection
Pipox	Pipecolic acid oxidase	-/-	15.8 ± 0.2	3	16.1 ± 0	3	Enzyme on pathway reactions
Plk1	Polo-like kinase 1, serine/threonine protein kinase	+/-	16.1 ± 0	3	16 ± 0.2	3	Random selection
Pmm2	Phosphomannomutase 2	+/-	16 ± 0.1	3	16.2 ± 0.1	3	Random selection
Ptpn12	Protein tyrosine phosphatase, non-receptor type 12	+/-	15.9 ± 0.1	3	15.7 ± 0.2	3	Random selection
Pttg1	Pituitary tumor-transforming gene 1	-/-	16.1 ± 0	3	15.8 ± 0.2	3	Random selection
Rock1	Rho-associated coiled-coil containing protein kinase 1	+/-	16 ± 0.2	3	16.1 ± 0.2	3	Random selection
Sra1	Steroid receptor RNA activator 1	-/-	16.2 ± 0.1	3	15.9 ± 0	3	Random selection
Ulk3	Unc-51-like kinase 3	-/-	16.1 ± 0.2	3	16 ± 0.2	3	Random selection
Ywhaz	Tyrosine 3-monooxygenase/tryptophan 5-monooxygenase activation protein	+/-	16.3 ± 0.1	3	16.4 ± 0.1	3	Enzyme on pathway reactions

Supplementary TableS2.Number of significantly changed metabolites by genotype effect and their alterations in 30 knockout strains.

Genotype	# of Significant metabolites	Significant clusters	FC > 2 or FC < 0.5	FC > 5 or FC < 0.2	FC > 10 or FC < 0.1
A2m-/-	201	13	36	0	2
Ahcy+/-	128	10	22	2	0
Atp5b+/-	181	13	52	3	1
Atp6v0d1+/-	120	10	29	2	0
C8a-/-	203	14	50	1	3
Cdk4+/-	135	16	29	3	1
Dhfr+/-	184	13	43	5	1
Dync1li1+/-	229	13	63	7	0
G6pd2-/-	113	8	26	5	4
Galc+/-	154	9	36	2	2
Gnpda1+/-	143	9	29	2	2
Idh1-/-	156	13	28	4	0
Iqgap1-/-	137	13	17	1	2
Lmbrd1+/-	198	13	53	2	1
Mfap4-/-	192	10	34	4	1
Mmachc+/-	182	13	35	2	3
Mvk+/-	230	13	43	9	4
Nek2-/-	205	15	47	1	3
Npc2+/-	213	14	64	7	2
Pebp1-/-	183	13	36	4	3
Phyh-/-	159	8	39	5	5
Pipox-/-	121	5	22	2	1
Plk1+/-	127	10	27	3	2
Pmm2+/-	232	18	59	6	7
Ptpn12+/-	149	7	32	2	2
Pttg1-/-	179	17	38	5	1
Rock1+/-	97	7	26	2	1
Sra1-/-	181	15	33	3	2
Ulk3-/-	213	17	64	8	2
Ywhaz+/-	154	12	36	7	7

Supplementary Table S3. Examples of sexual dimorphism of phenotypes (continuous traits) in wildtype mice and their alterations in 30 knockout lines.

Phenotype	Sex Effect in WT	Statistical Analysis in KO Lines	
		Genotype Effect	Genotype-sex Interaction
Heart weight, mg	***	A2m, Atp5b, Atp6v0d1, Cdk4, Gnpda1, Iqgap1	A2m, Atp6v0d1, Iqgap1
Body weight, g	***	A2m, Atp6v0d1, C8a, Cdk4, Dhfr, Dync1li1, Idh1, Iqgap1, Npc2, Pebp1, Plk1, Sra1	A2m, C8a, Dync1li1, Npc2, Pebp1, Sra1
Heart/Body weight, mg/g	ns	Atp5b, Atp6v0d1, C8a, Idh1, Mvk, Nek2, Pipox, Ulk3, Ywhaz	C8a, Pipox, Ywhaz
Alkaline phosphatase	***	Dhfr, Gnpda1, Plk1, Sra1, Ulk3	Dhfr, Sra1, Ulk3
Total cholesterol	***	Atp6v0d1, C8a, Dync1li1, Galc, Gnpda1, Idh1, Sra1, Ulk3	Galc
HDL cholesterol	***	A2m, Atp6v0d1, Galc, Gnpda1, Lmbrd1, Mfap4, Mmachc, Pebp1	Galc, Lmbrd1, Mmachc, Pebp1
Non-HDL cholesterol [a]	ns	Ahcy, C8a, Dhfr, Galc, Gnpda1, Idh1, Npc2, Rock1, Sra1, Ulk3	C8a, Galc, Idh1, Ulk3
Triglycerides	***	A2m, Ahcy, Dync1li1, Lmbrd1, Npc2<br>A2m, Ahcy, Dync1li1, Lmbrd1, Mfap4, Mvk, Npc2, Pipox	Ahcy, Npc2
Tibia length, mm	ns	Atp5b, Atp6v0d1, Cdk4, Dhfr, Dync1li1, G6pd2, Idh1, Iqgap1, Lmbrd1, Mfap4, Mmachc, Nek2, Phyh, Pipox, Plk1, Pmm2, Pttg1, Rock1, Sra1, Ywhaz	Atp5b, Dhfr, Dync1li1, Mfap4, Mmachc, Phyh, Pipox, Plk1, Pttg1, Sra1
Heart/Tibia length, mg/cm [a]	***	Ahcy, Atp5b, Atp6v0d1, Cdk4, Dhfr, G6pd2, Idh1, Iqgap1, Lmbrd1, Mfap4, Mmachc, Phyh, Pipox, Plk1, Pmm2, Ptpn12, Sra1, Ywhaz	Dhfr, Pipox, Pmm2, Sra1

[a] Values were additionally calculated from measured phenotypes.

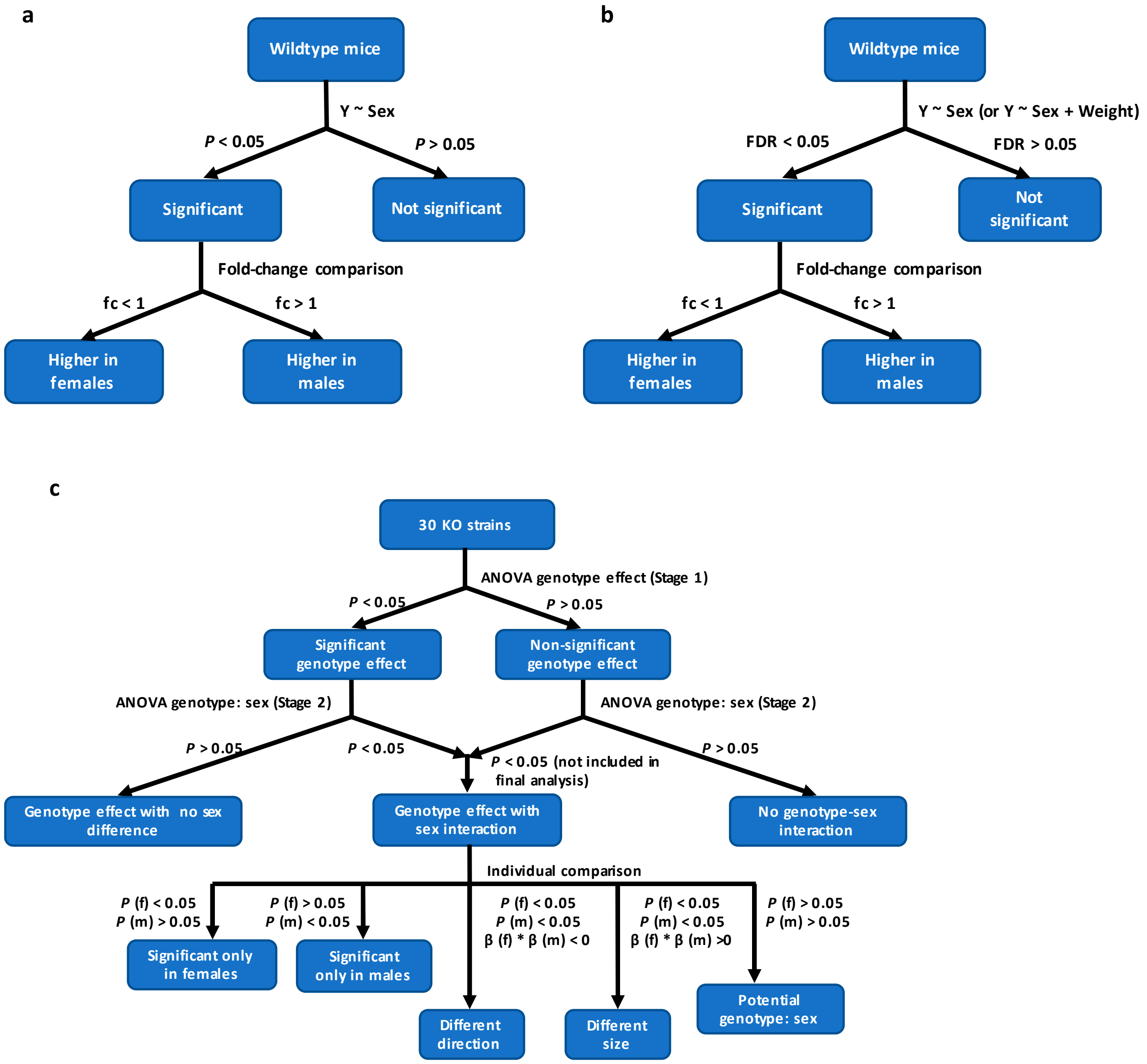

Supplementary Figure S1. Statistical analysis designs to study sexual dimorphism in wildtype mice and in knockout mice.

(a) Statistical analysis in wildtype mice using generalized linear model at $P < 0.05$

(b) Statistical analysis in wildtype mice using generalized linear model at $P < 0.05$ with false discovery rate correction (FDR < 0.05) and body weight adjustment

(c) Statistical analysis for genotype-sex interaction effect in knockout lines compared to wildtype mice using two-way ANOVA followed by individual comparison by generalized linear model at $P < 0.05$.

Statistical tests were adopted from Karp, N.A.; Heller, R.; Yaacoby, S.; White, J.K.; Benjamini, Y. Improving the Identification of Phenotypic Abnormalities and Sexual Dimorphism in Mice When Studying Rare Event Categorical Characteristics. Genetics 2017, 205, 491–501

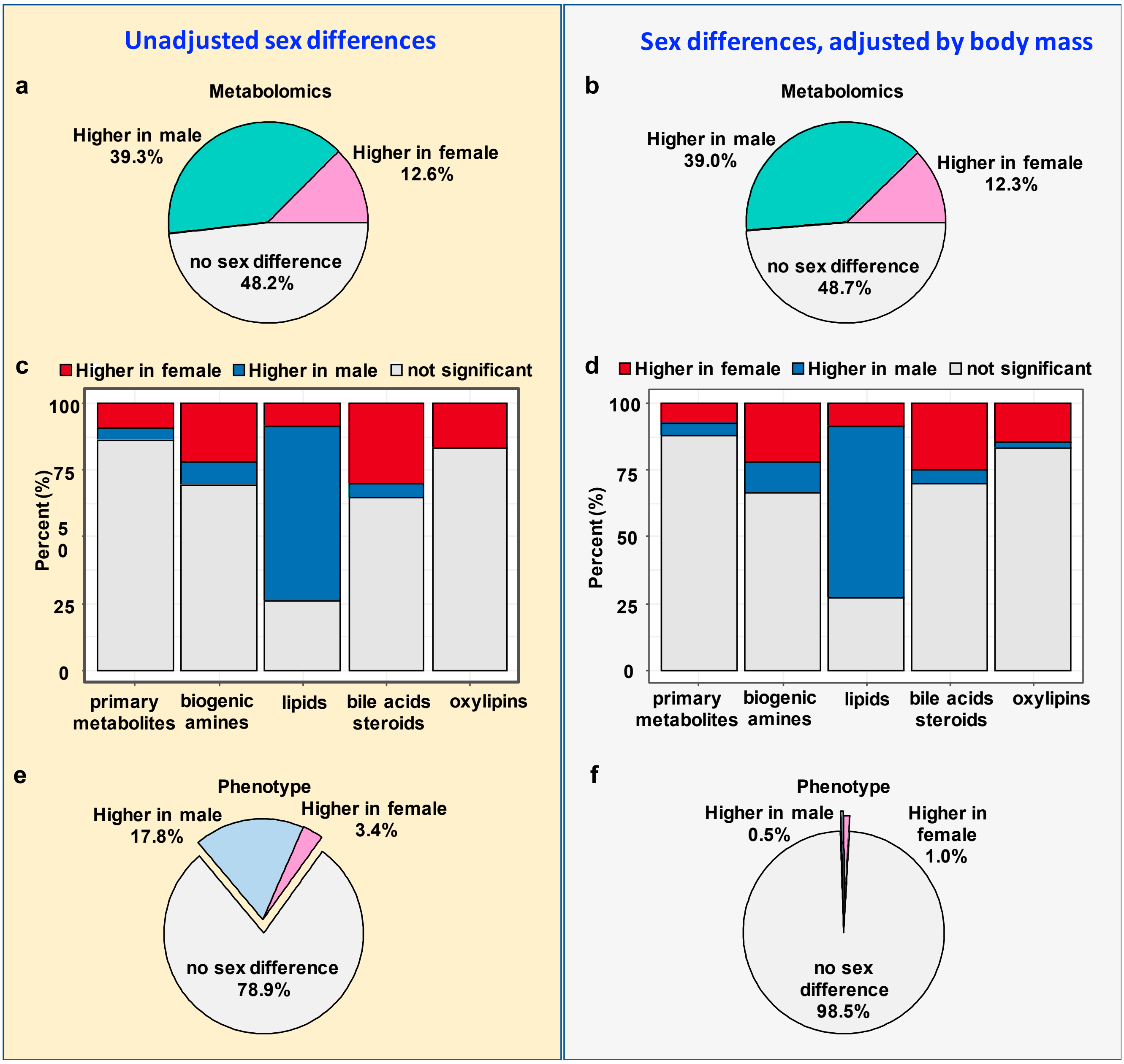

Supplementary Figure S2. Sex as a biological variable in metabolomics data with a stricter significance threshold and adjusted to body mass in wildtype C57BL/6NCrl mice (n = 20 males & 20 females, generalized linear model at FDR < 0.05).

(a) Proportion of metabolites significantly affected by sex

(b) Proportion of metabolites significantly affected by sex, adjusted by body mass.

(c) Assay-based proportion of sex-affected metabolites.

(d) Assay-based proportion of sex-affected metabolites,
adjusted by body mass.

(e) Proportion of IMPC phenotypes of continuous traits significantly affected by sex.

(f) Proportion of IMPC phenotypes of continuous traits significantly affected by sex; adjusted by body mass.

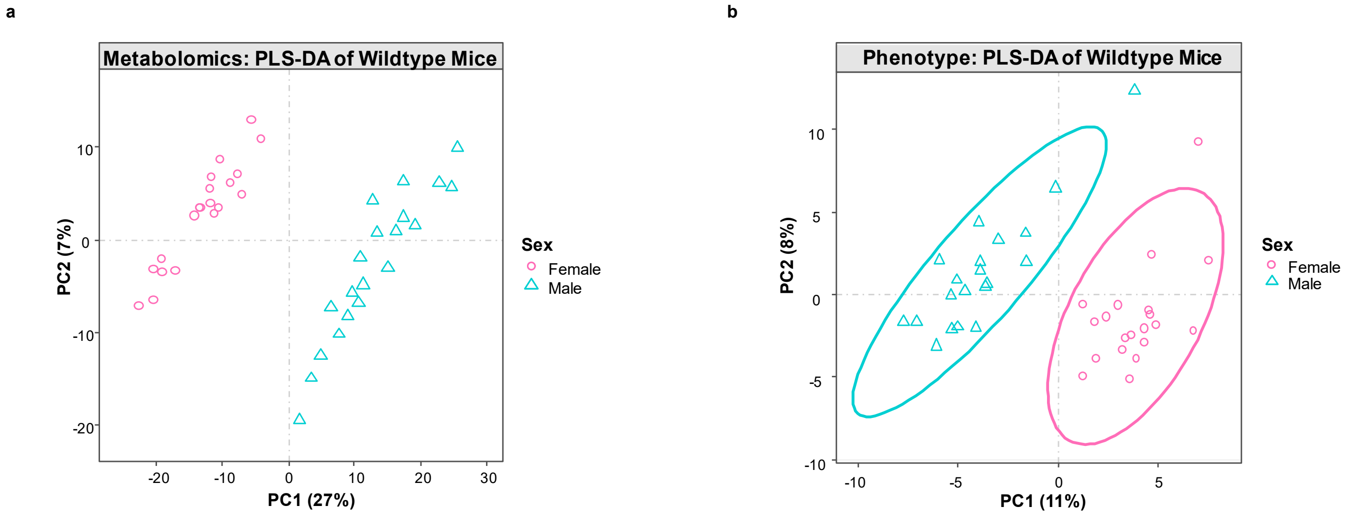

Supplementary Figure S3.

Sexual dimorphism of metabolomics data and phenotype data in wildtype mice (n = 20 males & 20 females).

(**a**) PLS-DA plot of metabolomics data in wildtype mice.

(**b**) PLS-DA plot of IMPC phenotype data in wildtype mice.

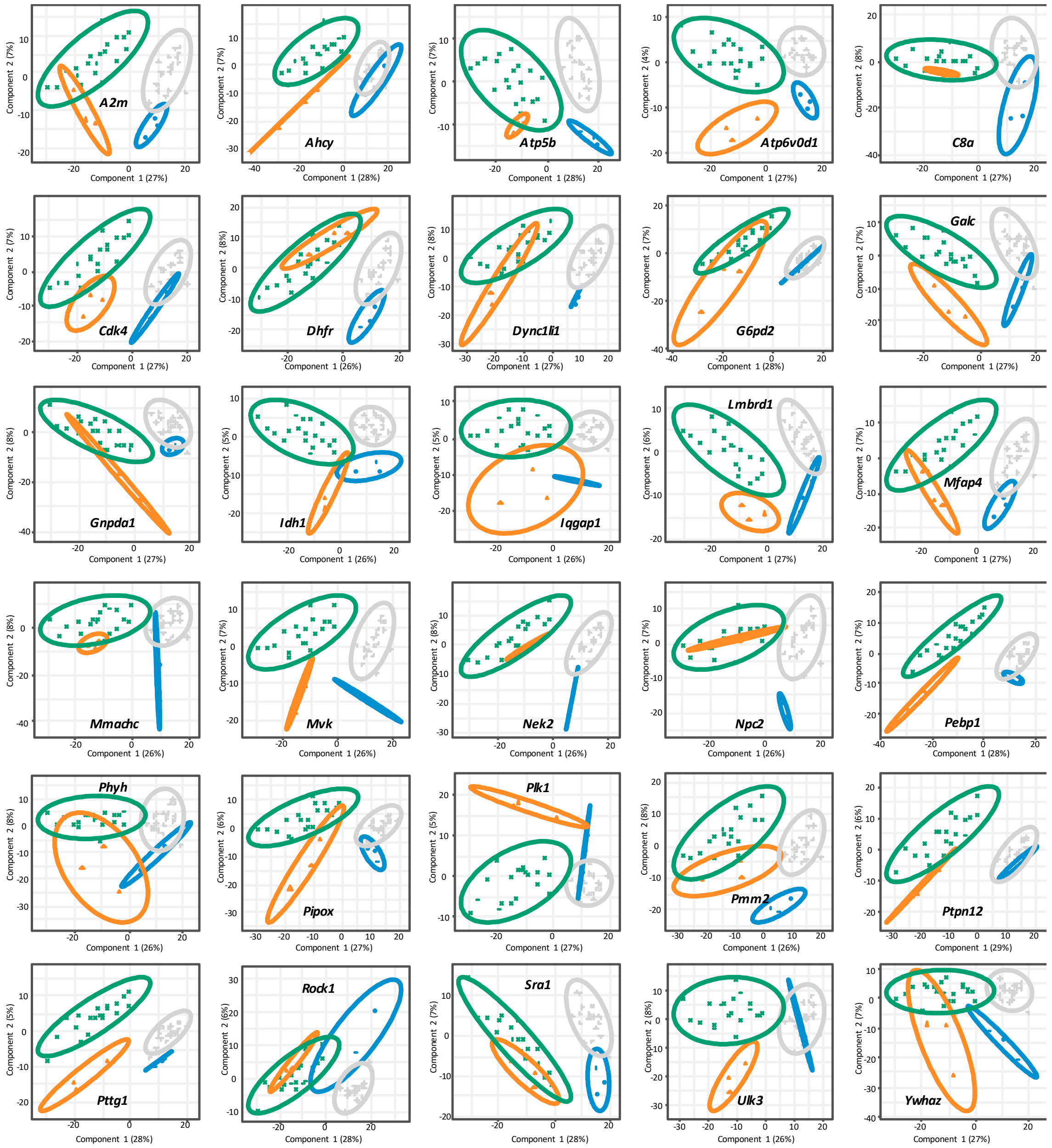

Group

- O KO Female
- △ KO Male
- + WT Female
- X WT Male

Supplementary Figure S4. PLS-DA plots of metabolomic data in 30 KO mouse lines compared to wildtype mice (n = 20 males & 20 females in C57BL/6NCrl wildtype mice, n = 3 male & 3 female mice in each KO mouse line). Circles: confidence intervals for plasma metabolic phenotype variance. Axes: Regression vectors with % of total explained variance.

(5a)

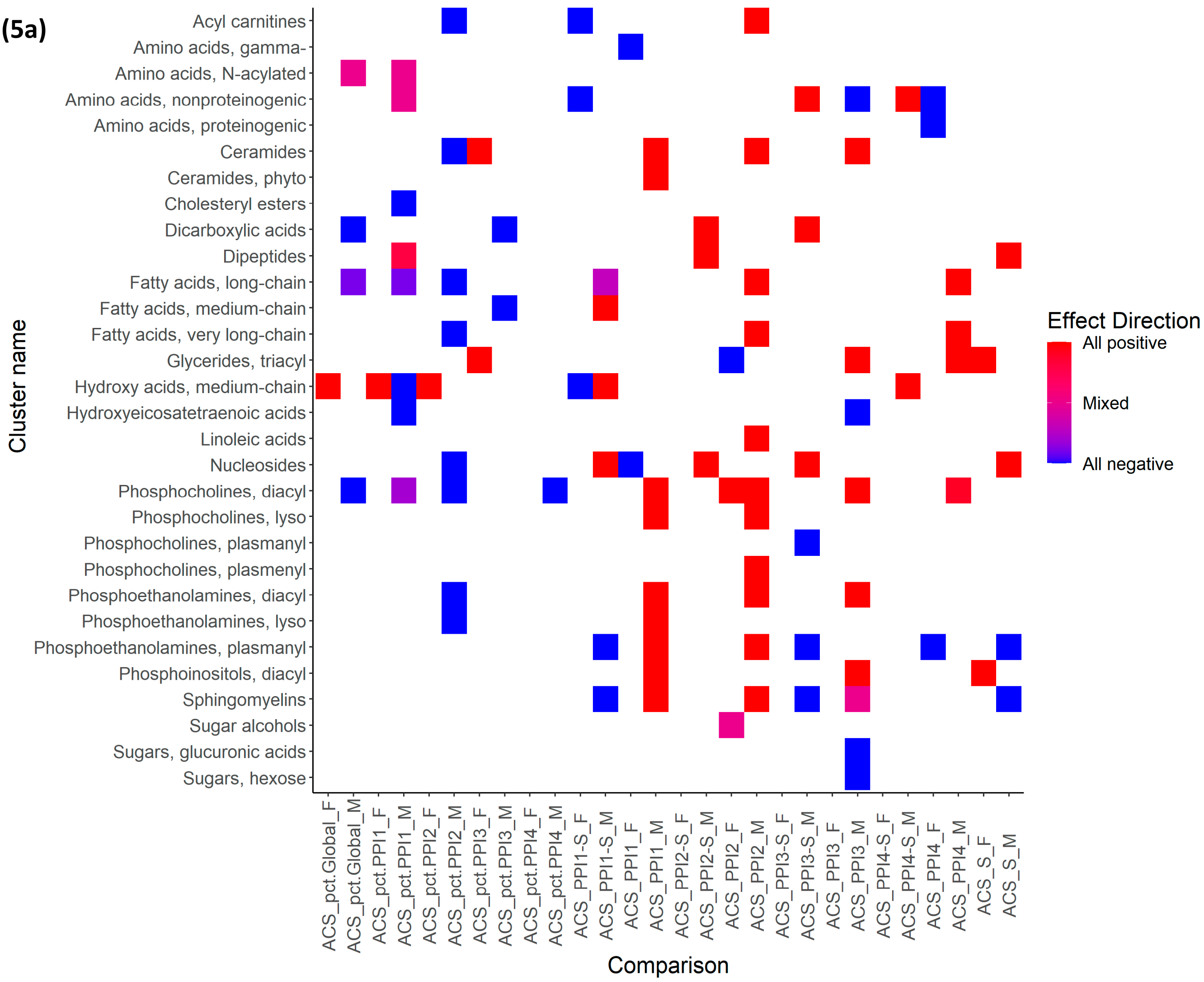

Supplementary Figure S5a

Clusters of significant phenotype associations between metabolites and Acoustic Startle and Pre-pulse Inhibition (PPI, week 10).

The acoustic startle response is characterized by an exaggerated flinching response to an unexpected strong auditory stimulus (pre-pulse). This response can be attenuated when it is preceded by a weaker stimulus (pre-pulse) and is the principle underlying pre-pulse inhibition (PPI). Several clinical studies have shown that a number of human disorders have impaired PPI including: schizophrenia, Huntington’s disease, fragile X syndrome, and autism. Heatmaps of ChemRICH set enrichment clusters for female (F) and male (M) wildtype mice, calculated from Spearman rank correlations of metabolite versus IMPC phenotypes.

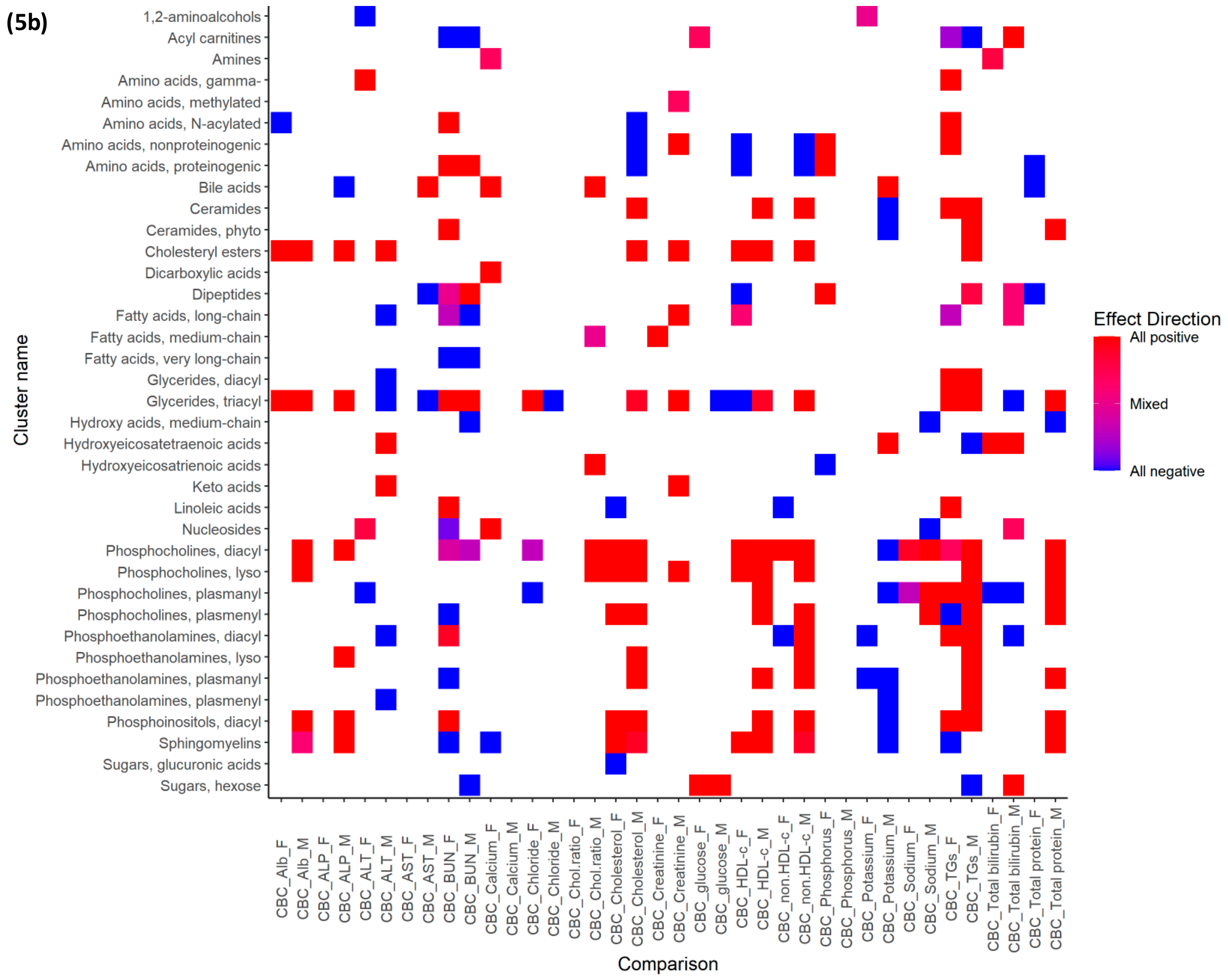

Supplementary Figure S5b

Clusters of significant phenotype associations between metabolites and Clinical Chemistry (CBC, week 16).

Clinical chemistry determines biochemical parameters in plasma including enzymatic activity, specific substrates and electrolytes. Heatmaps of ChemRICH set enrichment clusters for female (F) and male (M) wildtype mice, calculated from Spearman rank correlations of metabolite versus IMPC phenotypes.

(5c)

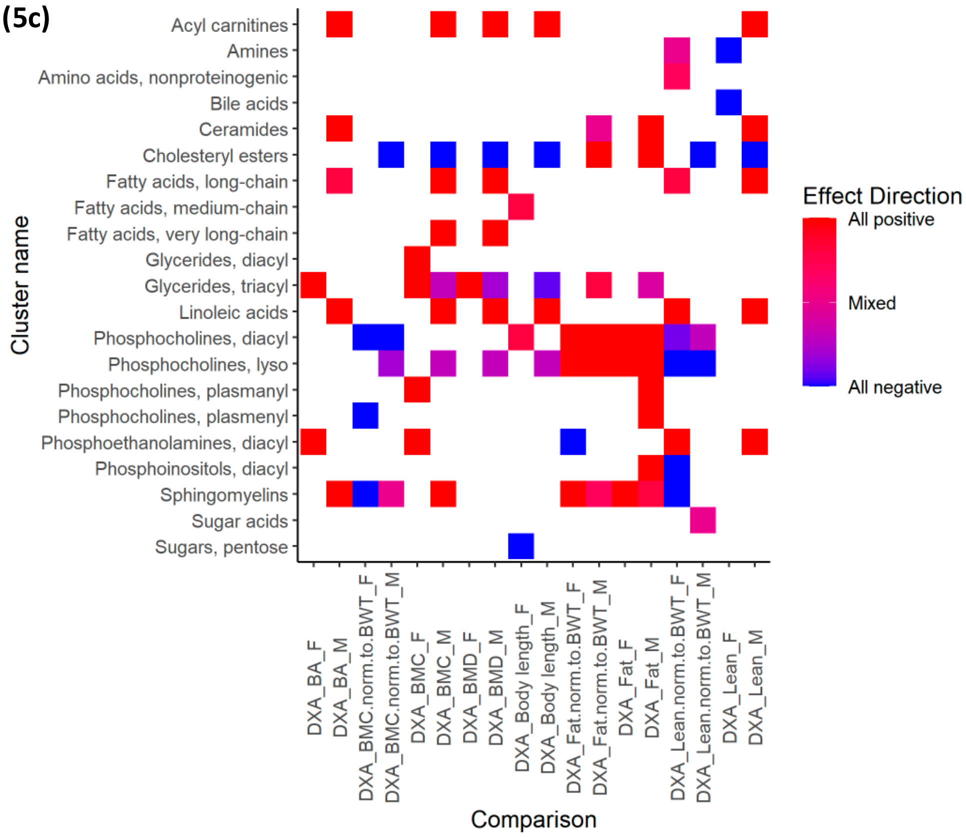

Supplementary Figure S5c

Clusters of significant phenotype associations between metabolites and Body Composition (DEXA lean/fat, week 14).

Measures of bone mineral content and density as well as body composition in mice using the DEXA (Dual Energy X-ray Absorptiometry) analyzer. Heatmaps of ChemRICH set enrichment clusters for female (F) and male (M) wildtype mice, calculated from Spearman rank correlations of metabolite versus IMPC phenotypes.

(5d)

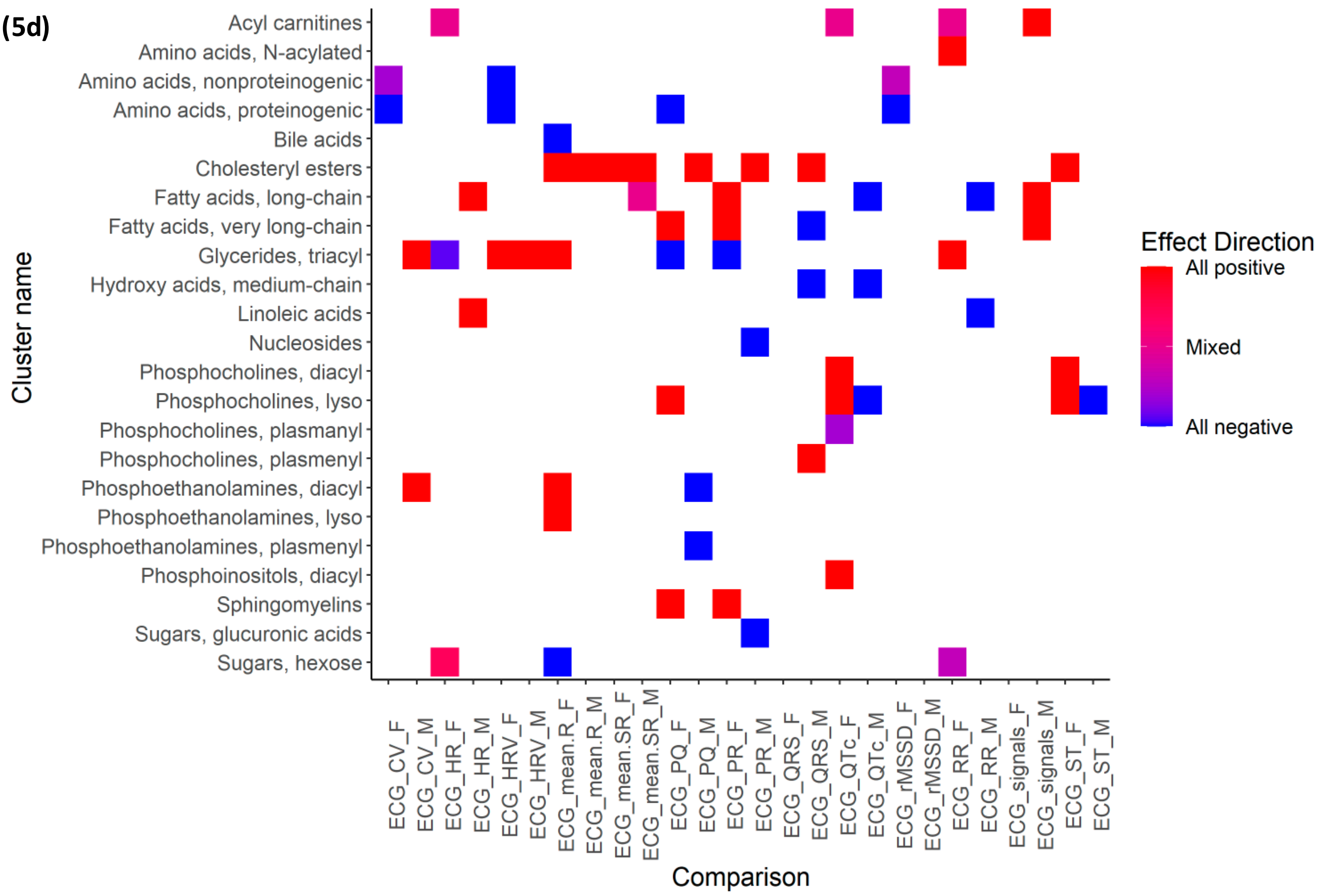

Supplementary Figure S5d

Clusters of significant phenotype associations between metabolites and Electrocardiogram (ECG, week 12).
Electrocardiographic recordings usually help to detect abnormal myocardial action potential, conduction of impulse, disturbances in cardiac rate and rhythm, and altered autonomic activities. Heatmaps of ChemRICH set enrichment clusters for female (F) and male (M) wildtype mice, calculated from Spearman rank correlations of metabolite versus IMPC phenotypes.

(5e)

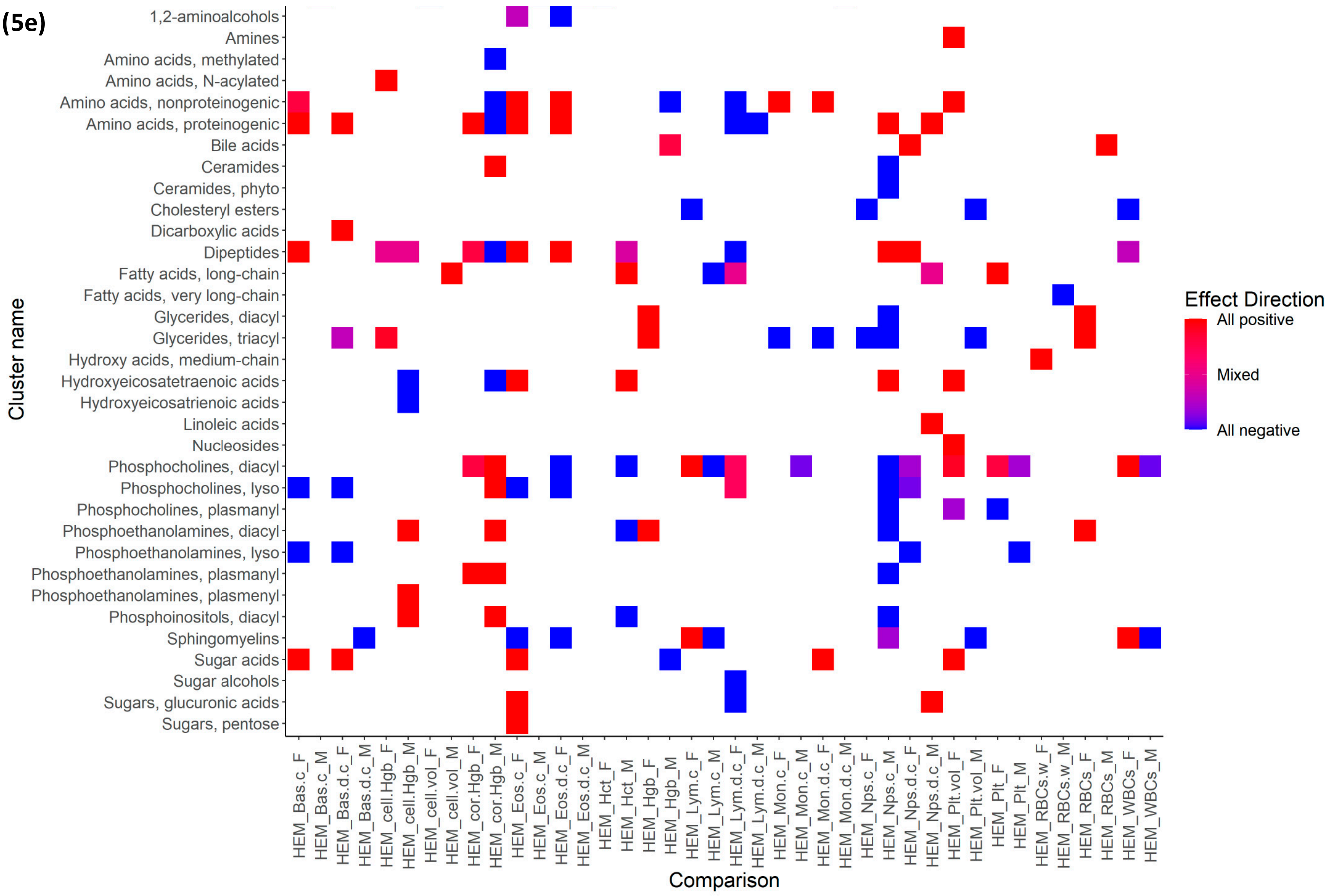

Supplementary Figure S5e

Clusters of significant phenotype associations between metabolites and Hematology (week 16).

Hematological assessment of blood determines blood cell counts (white blood cells, red blood cells, hemoglobin, and platelets) and additional hematological parameters (hematocrit, mean cell volume, mean corpuscular hemoglobin, mean cell hemoglobin concentration) can be derived using these indices. These tests will indicate abnormalities in the production of blood and its components (blood cells and hemoglobin) as well as in the associated blood-forming organs.

Heatmaps of ChemRICH set enrichment clusters for female (F) and male (M) wildtype mice, calculated from Spearman rank correlations of metabolite versus IMPC phenotypes.

(5f)

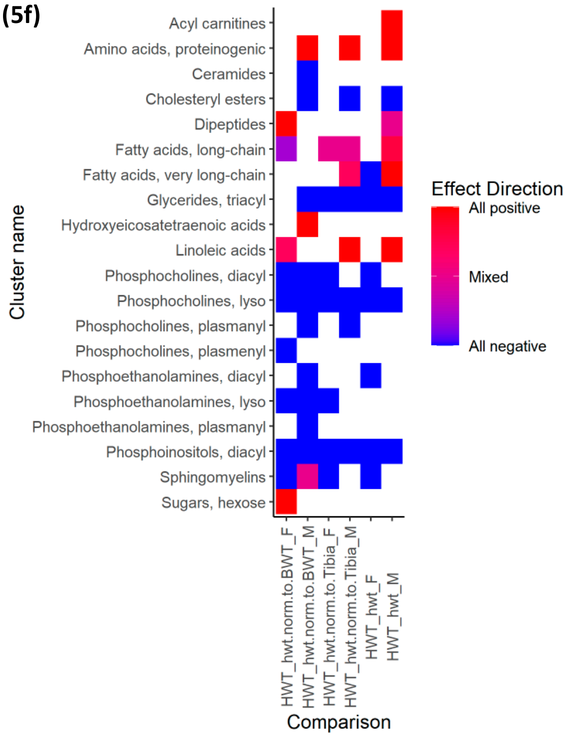

(5g)

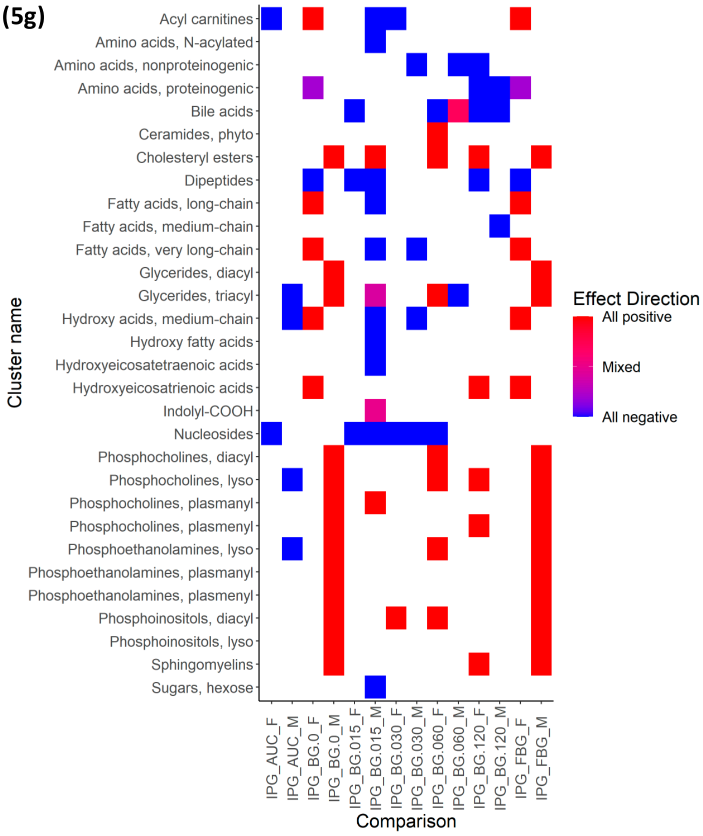

Supplementary Figure S5f

Clusters of significant phenotype associations between metabolites and heart weight (week 16).

To evaluate cardiac size using heart weight and body weight.

Heatmaps of ChemRICH set enrichment clusters for female (F) and male (M) wildtype mice, calculated from Spearman rank correlations of metabolite versus IMPC phenotypes.

Supplementary Figure S5g

Clusters of significant phenotype associations between metabolites and Intraperitoneal glucose tolerance (week 13).

The glucose tolerance test measures the clearance of an intraperitoneally injected glucose load from the body. It is used to detect disturbances in glucose metabolism that can be linked to human conditions such as diabetes or metabolic syndrome.

Heatmaps of ChemRICH set enrichment clusters for female (F) and male (M) wildtype mice, calculated from Spearman rank correlations of metabolite versus IMPC phenotypes.

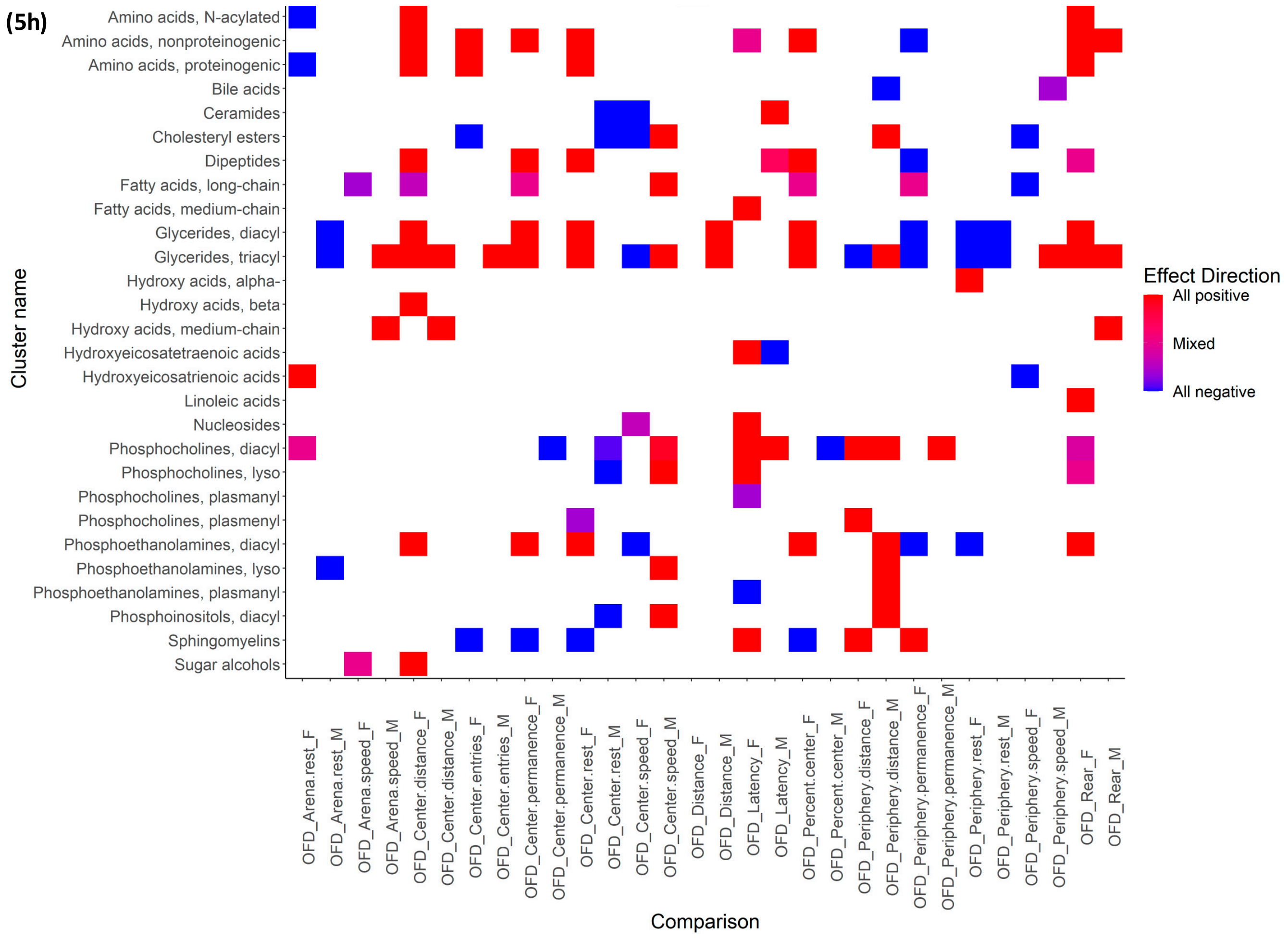

Supplementary Figure S5h

Clusters of significant phenotype associations between metabolites and Open Field (week 8).

The Open Field test is used to assess anxiety and exploratory behaviors. It is based on the natural tendency of an animal to explore and to protect itself using avoidance which translates to a normal animal spending more time in the periphery of the Open Field arena than in the center (the most anxiogenic area).

Heatmaps of ChemRICH set enrichment clusters for female (F) and male (M) wildtype mice, calculated from Spearman rank correlations of metabolite versus IMPC phenotypes.
